# Organic and inorganic–organic thin film structures by molecular layer deposition: A review

**DOI:** 10.3762/bjnano.5.123

**Published:** 2014-07-22

**Authors:** Pia Sundberg, Maarit Karppinen

**Affiliations:** 1 Department of Chemistry, Aalto University, P.O. Box 16100 FI-00076 Aalto, Finland

**Keywords:** atomic layer deposition (ALD), hybrid inorganic–organic thin films, molecular layer deposition (MLD), nanolaminates, nanostructuring, organic thin films, superlattices, thin-film technology

## Abstract

The possibility to deposit purely organic and hybrid inorganic–organic materials in a way parallel to the state-of-the-art gas-phase deposition method of inorganic thin films, i.e., atomic layer deposition (ALD), is currently experiencing a strongly growing interest. Like ALD in case of the inorganics, the emerging molecular layer deposition (MLD) technique for organic constituents can be employed to fabricate high-quality thin films and coatings with thickness and composition control on the molecular scale, even on complex three-dimensional structures. Moreover, by combining the two techniques, ALD and MLD, fundamentally new types of inorganic–organic hybrid materials can be produced. In this review article, we first describe the basic concepts regarding the MLD and ALD/MLD processes, followed by a comprehensive review of the various precursors and precursor pairs so far employed in these processes. Finally, we discuss the first proof-of-concept experiments in which the newly developed MLD and ALD/MLD processes are exploited to fabricate novel multilayer and nanostructure architectures by combining different inorganic, organic and hybrid material layers into on-demand designed mixtures, superlattices and nanolaminates, and employing new innovative nanotemplates or post-deposition treatments to, e.g., selectively decompose parts of the structure. Such layer-engineered and/or nanostructured hybrid materials with exciting combinations of functional properties hold great promise for high-end technological applications.

## Introduction

Many high-end technologies rely on our capability to fabricate thin films and coatings with on-demand tailored compositions and architectures in a highly controlled way. The atomic layer deposition (ALD) technique is capable of producing high-quality nanometer-scale thin films in an atomic layer-by-layer manner. Compared with other advanced gas-phase thin-film deposition techniques, ALD has several distinct advantages: The films can be deposited with a great control over the film thickness and they are not only pinhole free, dense and uniform, but also conformal even when deposited on complex three-dimensional (3D) structures. These features make ALD a method of choice for nanotechnology, for both material synthesis and device fabrication. The technology spectrum in which ALD can be utilized is extremely wide, including microelectronics, catalysis, energy applications and protective/barrier coatings.

**Figure 1:**
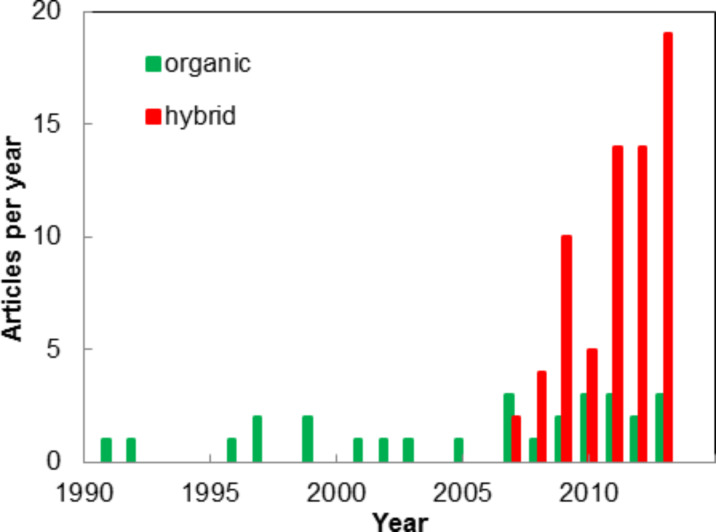
Number of articles annually published featuring organic and hybrid inorganic–organic thin films deposited by MLD and ALD/MLD.

The history of ALD goes back to the 1960s and 1970s [1–4]. Traditionally, ALD has been used to fabricate rather simple well-known inorganic materials, such as binary oxides and nitrides. The range of materials was fundamentally broadened by experiments producing organic polymers in the 1990s by a variant of ALD, now commonly known as molecular layer deposition (MLD), named after the molecular layer-by-layer fashion the film grows during the deposition [5–9]. Then – most excitingly – in the late 2000s the two techniques, ALD and MLD, were combined to produce inorganic–organic hybrid materials (Figure 1), making it possible to synthesize totally new material families with versatile characteristics, which are not accessible by any other existing technique [10–14].

**Figure 2:**
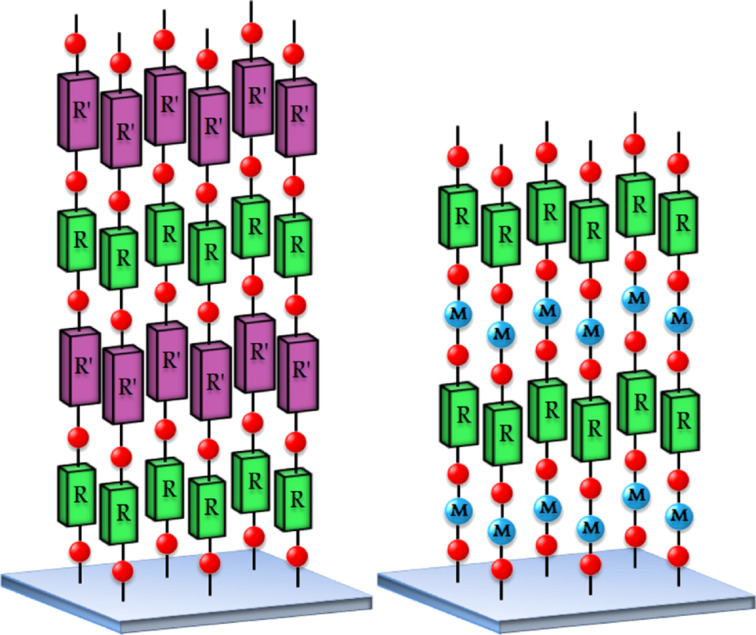
Schematic illustration of purely organic thin films grown by MLD (left) and hybrid inorganic–organic thin films grown by ALD/MLD (right).

In the combined ALD/MLD process organic molecules are covalently bonded to the metal atoms and vice versa, forming periodic thin-film structures that can be imagined to consist of either interlinked hybrid inorganic–organic polymer chains of essentially identical lengths or alternating two-dimensional (2D) planes of inorganic and organic monolayers (Figure 2). The hybrid thin films may not only possess properties combined from those of the two parent materials, but may also have completely new material properties, making them excellent candidates for a wide range of applications. Possible uses for the hybrid ALD/MLD films include optoelectronic devices, sensors, flexible electronics, solar cell applications, and protective coatings, to name only a few. It is also straightforward to make porous structures from the ALD/MLD grown hybrids by removing the organic part by simple annealing or wet-etching procedures [15–16]. Further tuning of material properties may be achieved by combining different inorganic, organic and hybrid layers into various thin-film mixtures, superstructures and nanolaminates. For example, precise control of the refractive index is extremely important in optical applications [17], while control of the electrical properties is required for storage capacitors, non-volatile memories as well as for transparent thin-film transistors [18–19]. Moreover, the tunability of the surface roughness is advantageous when fabricating gas sensors [20].

Over the years a number of excellent reviews featuring various types of ALD processes have been published, most recently, e.g., by Puurunen [4], George [21] and Miikkulainen et al. [22]. A review by Knez et al. [23] focuses on nanostructure fabrication by ALD. Although the introduction of the MLD method dates back two decades, the number of articles featuring purely organic thin films is still quite limited. Nevertheless some reviews concerning MLD-based thin films have been published in the past: George et al. [24] discuss the surface chemistry of MLD grown materials, addressing the problems which arise when using organic precursors in the growth process; Leskelä et al. [25] shortly review the novel materials fabricated by ALD and MLD; George [26], George et al. [27] and Lee et al. [28] focus on metal alkoxide thin films; Yoshimura et al. [29] discuss a possibility to utilize MLD in cancer therapy applications; King et al. [30] describe fine particle functionalization by ALD and MLD; and the review by Zhou et al. [31] covers all the organic interfaces fabricated by MLD.

The aim of this review is to provide a thorough investigation of the various thin films deposited by taking advantage of the currently strongly emerging MLD technique, including pure organic thin films, hybrid inorganic–organic thin films and their mixtures and nanolaminate structures. First we will describe the sequential ALD/MLD process, followed by a few words about the organic precursors used in these processes. Then all the various materials fabricated utilizing MLD are reviewed: The purely organic materials are summarized first and the inorganic–organic materials are discussed in a separate chapter. Lastly, the variously mixed and nanostructured ALD/MLD and MLD materials are presented.

## Review

### Deposition cycle and ideal ALD/MLD growth

**Figure 3:**
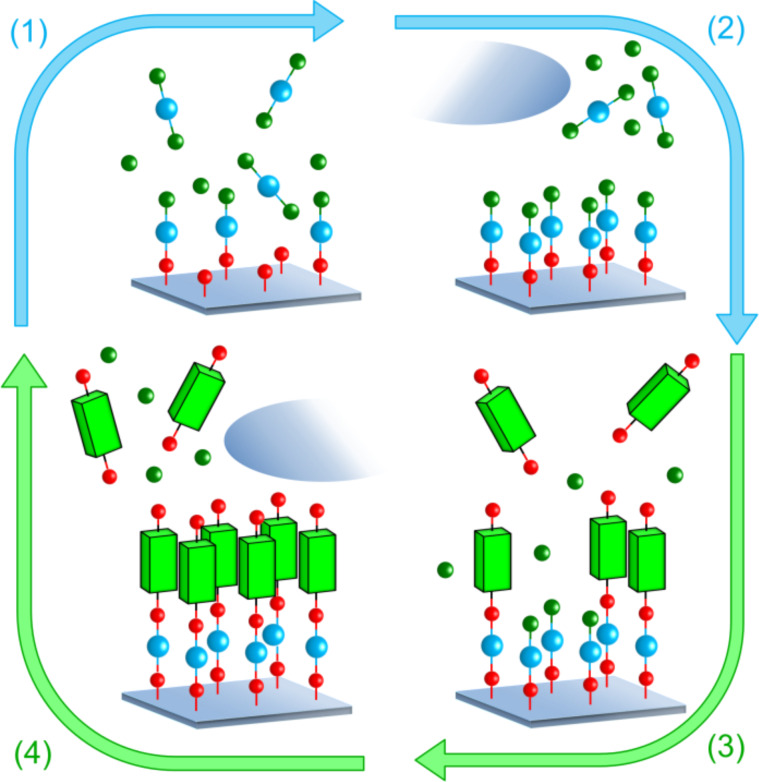
An ALD/MLD cycle consisting of the following four steps: (1) the first (inorganic) precursor is pulsed to the reactor and it reacts with the surface species, (2) the excess precursor and possible byproducts are removed from the reactor, either by purging with inert gas such as nitrogen or argon, or by evacuation, (3) the second (organic) precursor is pulsed to the reactor and it reacts with the surface species, and finally (4) the excess precursor/possible byproducts are removed from the reactor. In an ideal case a monolayer of a hybrid inorganic–organic material is formed. To deposit thicker films this basic ALD/MLD cycle is repeated as many times as needed to reach the targeted film thickness.

In both ALD and MLD the gas–solid reactions occur in a self-limiting, surface-saturated manner. The characteristic ALD/MLD growth can be described by a so-called ALD and/or MLD cycle. The number of precursors employed during an ALD or MLD process can be varied, but a prototype process is based on two. For example, in case of the hybrid inorganic–organic films one inorganic and one organic precursor are used, and the ALD/MLD cycle can be separated into four steps consisting of precursor pulsing and intermediate purging steps as described in Figure 3.

To ensure the self-limiting growth both the precursor pulsing and purging steps should be sufficiently long. In an ideal process the surface is fully covered with the precursor in each precursor pulsing step, but in practice only a partial coverage is typically achieved. The so-called growth-per-cycle (GPC) value is the average increase in film thickness during one ALD/MLD cycle.

When the GPC remains constant with increasing number of deposition cycles, the growth is said to be linear. In some cases, however, the GPC is not constant from the beginning. The substrate may inhibit or enhance the film growth depending on the compatibility of chemistries of the substrate surface and the growing film, in which case the GPC is initially lower or higher before settling to a constant value [4]. It is the sequential self-limiting nature of ALD and MLD that enables the great thickness control and conformal growth of the films, which in turn makes the two techniques, ALD and MLD, and their combinations such a great asset for nanotechnology.

**Figure 4:**
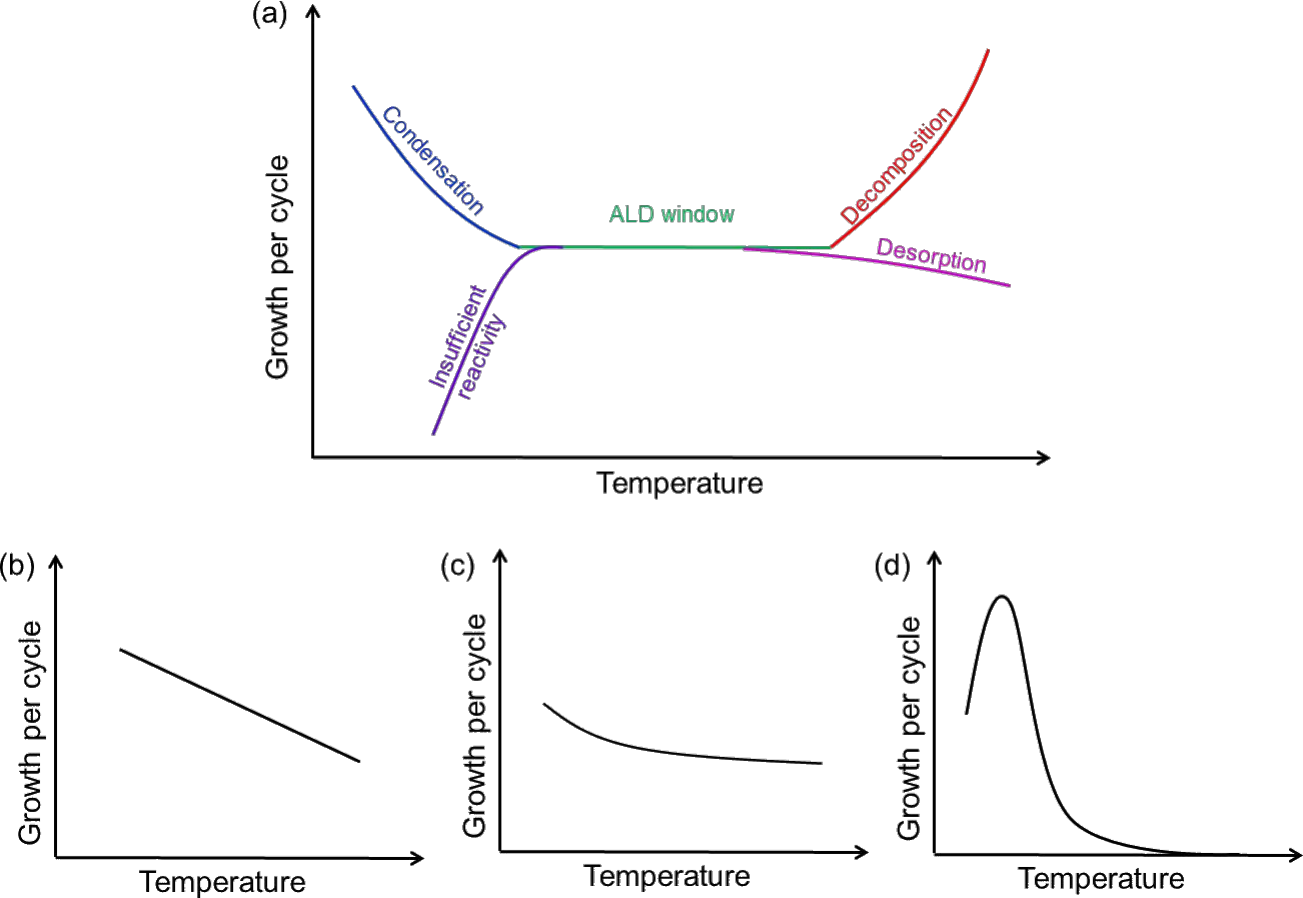
Dependence of the film growth on the deposition temperature: (a) within the so-called ALD window the growth per cycle remains constant with increasing temperature, whereas (b)–(d) represent typical cases in which no temperature range of constant growth is seen, but the process may yet be highly reproducible.

The ALD or MLD growth typically depends on the deposition temperature at least in some temperature ranges. The effect of temperature on the GPC value is often described by a concept known as an ALD (or MLD) window (Figure 4a). The ALD window has been defined as a regime in which the GPC remains constant and does not depend on process parameters like temperature, gas pressure, precursor flows or purging times. Outside the ALD window the GPC value may be higher due to precursor condensation (at too low deposition temperatures) or decomposition (at too high deposition temperatures), whereas limited growth may result from insufficient reactivity (at too low deposition temperatures) or desorption (at too high deposition temperatures) of the precursor. However, the existence of an ALD window is not a necessary prerequisite for an ALD-type growth, and such a window is not found for all well-behaving ALD (or MLD) processes. Examples of typical cases in which no temperature range of constant growth is seen, but the process may yet be highly reproducible are shown in Figure 4b–d. The growth may occur in the way shown in Figure 4b when the growth is not fully of ALD type, but one of the precursors diffuses into the film, improving the growth by providing more reactive sites: The diffusion out of the film is enhanced at higher temperatures, resulting in a lower growth rate [12]. The decrease in growth at increasing deposition temperatures may be observed in general when the temperature affects the number of reactive sites or the reaction mechanism. The combined effect of reaction activation (increase in growth), followed by decrease on reactive sites may result in a growth such as shown in Figure 4d [4].

**Figure 5:**
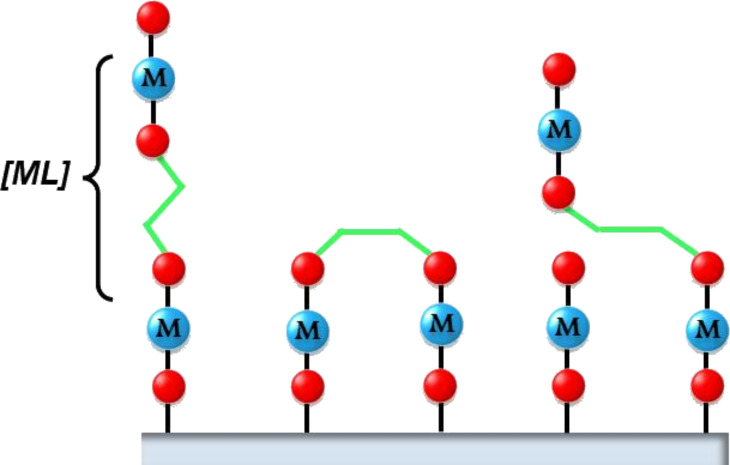
Ideally, the organic precursor molecule reacts with one surface site only and remains straight (left). It may also react twice with the surface (middle) or tilt (right).

The MLD and ALD/MLD films often show lower than anticipated GPC values. There are several possible causes for the hindered growth (Figure 5). Organic precursor molecules with long chains are likely to tilt such that the growth is not perfectly perpendicular to the surface. Likewise, organic molecules may bend and react twice with the surface, reducing the number of reactive surface sites and lowering the growth rate. Organic precursors are also often bulky, causing steric hindrance. The various difficulties encountered when using organic precursors are discussed in detail in a review by George et al. [24]. Several strategies have been employed to improve the controllability of the growth process, such as using organic precursors with stiff backbones [13–14,32–37] or with two different functional groups [36–39], using reactions requiring surface activation [10,39–46], using precursors in which ring-opening reactions occur [47], or using three different precursors instead of two [48–49].

The ratio, *r* = *GPC*/*ML*, where *ML* is the ideal length of the M–R monomer (without bending, see Figure 5), provides us with a measure of the perfectness of the growth. Thus, ideally *r* = 1. The *r* value achieved varies greatly with different precursor combinations. For purely organic MLD films *r* is typically lower than 0.5, exceptions being the hexanedioyl dichloride+hexane-1,6-diamine [8,50] and heptane-1,7-diamine+nonanedioyl dichloride [7] systems. For hybrid ALD/MLD thin films there is a larger variation in the *r* values depending on the organic precursor employed. The choice of the metal precursor seems to have a significant effect, too. For example, with the linear ethane-1,2-diol (ethylene glycol, EG) molecule as the organic precursor and trimethylaluminum (Al(CH_3_)_3_, TMA), diethylzinc (Zn(C_2_H_5_)_2_, DEZ), titanium tetrachloride (TiCl_4_) and zirconium *tert*-butoxide (Zr(CH_4_H_9_O)_4_, ZTB) as the inorganic precursors the growth processes have yielded *r* values of 0.6 0.1, 0.6 and 0.2, respectively [12,51–54]. In case of the aromatic precursor benzene-1,4-diol (hydroquinone, HQ), *r* values of 0.4 and 0.2 have been achieved for Al-based [13] and Zn-based [33] films, respectively. Systems exhibiting *r* values close to unity have been reported, such as TiCl_4_+4-aminophenol [37] and hexa-2,4-diyne-1,6-diol (HDD) [55–56] containing hybrid films. In case of the TiCl_4_+4-aminophenol process, the excellent growth could be attributed to the two different functional groups and the stiff aromatic backbone of 4-aminophenol as well as to the small –Cl ligands in TiCl_4_ [37]. The HDD molecule with two triple bonds is also stiff and during the deposition process also the formation of bridging alkanes is induced by UV radiation after precursor pulsing steps [55–56]. However, as the hybrid systems seem to be sensitive regarding the process parameters, especially considering pulsing and purging times, there are systems with *r* values which are considerably higher than 1, e.g., TMA+heptanedioic acid [57] and TMA+oxiran-2-ylmethanol (glycidol, GLY) [38].

The characterization techniques used to investigate the thin films deposited by using MLD do not vary much from those techniques used for inorganic thin films grown by ALD. An in situ quartz crystal microbalance (QCM) is often used to give some insight on the growth dynamics of the deposition. Besides thickness measurements, X-ray reflectivity (XRR) can be used for evaluating densities and roughnesses of the thin films. The crystallinity of the films is examined by X-ray diffraction (XRD). The topography of the films can be investigated by using atomic force microscopy (AFM). Fourier transform infrared (FTIR) spectroscopy is useful for analyzing the chemical state of the films. The composition of the films can be studied by X-ray photoelectron spectroscopy (XPS), whereas the presence of a metal can be verified by X-ray fluorescence (XRF) measurements. Nanoindentation gives insight on the mechanical properties of the films.

### Organic precursors employed in MLD

Both ALD and MLD set some requirements for the precursors employed, such as sufficient vapor pressure, reactivity and stability at the reaction temperature, to ensure feasible film growth. Finding organic compounds which would fulfill these requirements is not straightforward. Many of the organic precursors exhibit low vapor pressures at room temperature and it is thus mandatory to heat them to achieve a sufficient precursor supply. In Table 1, we list all the organic compounds employed/investigated as precursors for ALD/MLD. Here it should be noted that organic compounds typically have several different names; the nomenclature we use is based on the recommendations of the International Union of Pure and Applied Chemistry (IUPAC), but in Table 1 commonly used other names for the compounds are also given. It should also be emphasized that not all the processes based on the precursors listed in Table 1 exhibit the characteristic features of an ALD/MLD process. In Table 1 we give – when accessible – the vapor pressures at 100 °C together with the temperatures corresponding to a vapor pressure of 2 mbar, which is a rather typical pressure used for ALD/MLD depositions.

**Table 1:** Organic compounds (and their different names and abbreviations) employed in MLD and ALD/MLD processes together with vapor pressures, *P*, at 100 °C and temperatures, *T*, corresponding to a vapor pressure of 2 mbar for some of the organic precursors (the values were calculated by using the equations and parameters obtained from the DIPPR Project 801 database (full version) [58]).

IUPAC name	abbreviation	names used in references	*P* (Pa)	*T* (°C)

		2,2’-(propane-2,2-diylbis(oxy))-diethanamine		
(1*E*)-prop-1-ene-1,2,3-tricarboxylic acid		*trans*-aconitic acid		
(2*E*,4*E*)-hexa-2,4-dienedioic acid		(2*E*,4*E*)-hexa-2,4-dienedioic acid; *trans*,*trans*-muconic acid		
(2S)-2-aminopentanedioic acid		L-glutamic acid		182
(*E*)-butenedioic acid		fumaric acid; (*E*)-butenedioic acid		165
(*Z*)-butenedioic acid		maleic acid; (*Z*)-butenedioic acid	6.37	142
1,2-bis[(diamethylamino)dimethylsilyl]ethane		1,2-bis[(diamethylamino)dimethylsilyl]ethane		
1,4-diaminobenzene		*p*-phenylenediamine; 1,4-phenylenediamine	154	104
1,4-diisocyanatobenzene	PDIC	1,4-phenylene diisocyanate		
1,4-diisocyanatobutane		1,4-diisocyanatobutane		
1,4-diisothiocyanatobenzene		1,4-phenylene diisothiocyanate		
2-aminoethanol		ethanolamine	6410	43
2-oxepanone		ε-caprolactone	1520	58
4,4’-oxydianiline	ODA	4,4’-oxydianiline; 4,4-diaminodiphenyl ether		
4-aminophenol	AP	4-aminophenol		
4-nitrobenzene-1,3-diamine		2,4-diaminonitrobenzene		
7-octenyltrichlorosilane	7-OTS	7-octenyltrichlorosilane		
8-quinolinol		8-hydroxyquinoline	199	100
benzene-1,2,4,5-tetracarboxylic acid		1,2,4,5-benzene tetracarboxylic acid; 1,2,4,5-benzotetracarboxylic acid		283
benzene-1,2-dicarboxylic acid		1,2-benzenedicarboxylic acid; 1,2-benzodicarboxylic acid		173
benzene-1,3,5-tricarboxylic acid		1,3,5-benzene tricarboxylic, 1,3,5-benzotricarboxylic acid		
benzene-1,3,5-triol		1,3,5-benzenetriol; phloroglucinol		
benzene-1,3-dicarboxylic acid		1,3-benzene dicarboxylic acid; 1,3-benzodicarboxylic acid		233
benzene-1,4-dicarboxylic acid		1,4-benzene dicarboxylic acid; 1,4-benzodicarboxylic acid		266
benzene-1,4-diol	HQ	1,4-benzendiol; hydroquinone	9.39	137
benzoic acid		benzoic acid	1820	71
but-2-yne-1,4-diol		2-butyne-1,4-diol	118	107
butane-1,4-diamine		1,4-butane diamine		
butanedioic acid		succinic acid	0.569	168
decane-1,10-diamine		1,10-diaminodecane		
decanedioic acid		decanedioic acid; sebacic acid	0.0466	198
decanedioyl dichloride		sebacoyl dichloride		
ethane-1,2-diamine	ED	ethylenediamine	56700	
ethane-1,2-diol	EG	ethylene glycol	2100	61
ethanedihydrazide		oxalic dihydrazide		
ethanedioic acid		ethanedioic acid; oxalic acid	50.9	117
ethanetetracarbonitrile		tetracyanoethylene		
furan-2,5-dione		maleic anhydride	3260	48
furo[3,4-f][2]benzofuran-1,3,5,7-tetrone	PMDA	pyromellitic dianhydride; 1,2,3,5-benzenetetracarboxylic anhydride		
heptane-1,7-diamine		1,7-diaminoheptane		
heptanedioic acid		heptanedioic acid; pimelic acid	0.806	175
hexa-2,4-diyne-1,6-diol	HDD	2,4-hexadiyne-1,6-diol; hexadiyne diol		
hexane-1,6-diamine		1,6-hexanediamine; 1,6-diaminohexane; hexamethylene diamine	3670	43
hexanedioyl dichloride		adipyl dichloride; adipoyl chloride		
*N*-(2-aminoethyl)ethane-1,2-diamine		diethylenetriamine	2610	52
*N*,*N*-bis(2-aminoethyl)ethane-1,2-diamine		triethylenetetramine	62.5	117
nonanedioyl dichloride		azelaoyl dichloride		
octane-1,8-diamine		1,8-diamino-octane		
octanedioic acid		octanedioic acid; suberic acid	0.109	185
octanedioyl dichloride		suberoyl dichloride		
oxiran-2-ylmethanol	GLY	glycidol	11400	27
pentanedioic acid		pentanedioic acid; glutaric acid	3.26	160
propane-1,2,3-tricarboxylic acid		tricarballylic acid		
propane-1,2,3-triol	GL	glycerol	25.6	131
propanedioic acid		propanedioic acid; malonic acid	4.55	147
propanedioyl dichloride		malonyl chloride		
terephthalaldehyde		terephthalaldehyde	320	94
terephthalic acid bis(2-hydroxyethyl) ester		terephthalic acid bis(2-hydroxyethyl) ester	0.00091	243
terephthaloyl dichloride		terephthaloyl dichloride; terephthaloyl chloride	172	103
tris(2-aminoethyl)amine		tris(2-aminoethyl)amine		
tris(2-hydroxyethyl)amine		triethanolamine	1.90	168

#### Organic thin films

So far MLD has been used to produce polyamide, polyimide, polyimide–amide, polyurea, polyurethane, polythiurea, polyester and polyimine thin films. In Table 2, we list the characteristic polymer linkages seen in these films. For example, polyamides are polymers in which the precursors employed are combined with each other via amide bond formation whereas polyureas contain the urea linkage. And like the name of polyimide–amides suggests, these polymers contain both an imide and an amide group. In the following sub-chapters all the different types of polymer thin films deposited by MLD until now are shortly presented.

**Table 2:** Characteristic linkages for the polymer types deposited using MLD.

polymer	characteristic linkage

polyamide	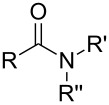
polyimide	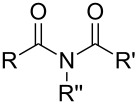
polyurea	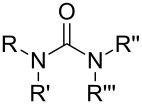
polyurethane	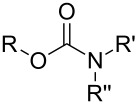
polythiourea	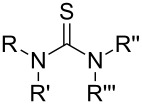
polyester	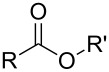
polyimine	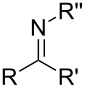

#### Polyamides

Polyamides are extremely durable and strong, making them useful materials for a wide variety of applications, e.g., textiles, automotive industry applications and electronics. Polyamides can be classified to be aliphatic, semi-aromatic or aromatic, depending on the composition of the polymer chain. The polyamides produced by MLD have been deposited by using diamines and acyl dichlorides as precursors (Table 3). The majority of these polyamides are aliphatic; so far only one semi-aromatic and one aromatic polyamide have been fabricated.

**Table 3:** Acyl dichloride and diamine precursors used to fabricate polyamide thin films by MLD.

precursor A	precursor B	references

hexanedioyl dichloride	hexane-1,6-diamine	[8,50]
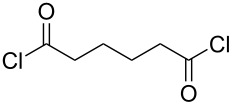	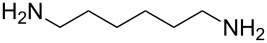	
nonanedioyl dichloride	heptane-1,7-diamine	[6]
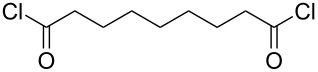	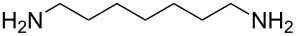	

hexanedioyl dichloride	hexane-1,6-diamine	[7]
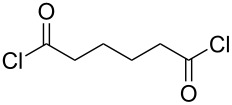	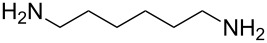	
octanedioyl dichloride	octane-1,8-diamine	
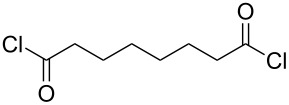	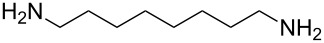	
decanedioyl dichloride	decane-1,10-diamine	
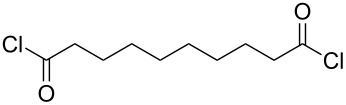	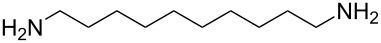	

terephthaloyl dichloride	butane-1,4-diamine	[59]
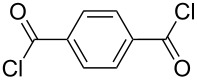	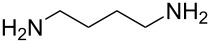	
terephthaloyl dichloride	1,4-diaminobenzene	[60]
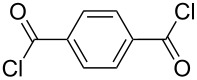	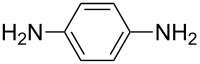	

Aliphatic polyamides, i.e., nylons, have been deposited by MLD from precursors with a wide range of chain lengths. The shortest monomers employed are hexanedioyl dichloride and hexane-1,6-diamine which have been used to make nylon 66 [8,50]. In the earlier work done by Shao et al. [8] the films were grown through polycondensation. The in situ FTIR studies by Du et al. [50] indicate that the deposition of nylon 66 displays characteristic ALD-type growth, i.e., linearity and self-limiting growth with the different precursor exposure lengths, although some ammonium chloride salt formation was observed. The highest GPC values for nylon 66 were 13.1 Å per cycle when deposited at 60 °C on pretreated Si(100) [8] and up to 19 Å per cycle on KBr substrates at the deposition temperature of 83 °C [50]. The latter value is somewhat higher than what the predicted unit-chain length, 17.4 Å, would suggest: This higher than predicted growth rate was attributed to a CVD-type growth. Kubono et al. [6] deposited nylon 79 from heptane-1,7-diamine and nonanedioyl dichloride at room temperature. The GPC value achieved, 18 Å per cycle, was quite close to the calculated length of the repeating unit of the polyamide, i.e., 22 Å. Nagai et al. [7] fabricated several series of different nylons, including systems where up to four precursors were used, but the growth rates of the films were not discussed.

Peng et al. [59] used butane-1,4-diamine and terephthaloyl dichloride to grow semi-aromatic polyamide thin films. The highest achieved growth rate of this type of polyamide on Si(100) substrates was only 2 Å per cycle at 85 °C. The near-edge X-ray absorption fine structure spectroscopy measurements showed that the oligomer units of the films were not perpendicular to the substrate surface but significantly tilted, suggesting that double reactions take place during the growth.

The only aromatic polyamide grown by MLD so far was fabricated by using terephthaloyl dichloride and 1,4-diaminobenzene as precursors. In industry, these two chemicals are used as precursors for a mechanically strong and thermally stable polymer known as Kevlar. The growth rate for the terephthaloyl dichloride*+*1,4-diaminobenzene MLD process was not constant: At 145 °C the measured GPC varied between 0.5 and 3.3 Å per cycle, which is considerably less than what could be assumed from the calculated chain length, which is 12.9 Å. The low growth rate was assumed to be due to the non-ideal polymer-chain orientation: rather than standing up the polymer chains were suggested to have a more parallel orientation towards the substrate. [60]

#### Polyimides

As with the polyamides discussed above, polyimides can be classified to be aliphatic, semi-aromatic or aromatic depending on the chain composition. Furo[3,4-*f*][2]benzofuran-1,3,5,7-tetrone (pyromellitic dianhydride, PMDA), widely used as a raw material for polyimides, has also been used as a precursor in all the MLD works on polyimide thin films so far (Table 4).

**Table 4:** Precursors used to deposit polyimide thin films.

precursor A	precursor B	precursor C	references

furo[3,4-*f*][2]benzofuran-1,3,5,7-tetrone	ethane-1,2-diamine		[61]
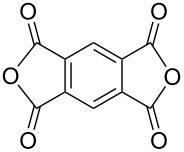	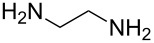		
furo[3,4-*f*][2]benzofuran-1,3,5,7-tetrone	hexane-1,6-diamine		[61–62]
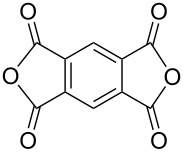	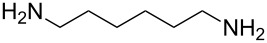		
furo[3,4-*f*][2]benzofuran-1,3,5,7-tetrone	4-nitrobenzene-1,3-diamine		[5]
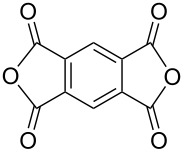	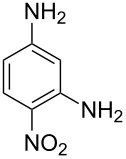		
furo[3,4-*f*][2]benzofuran-1,3,5,7-tetrone	4,4’-oxydianiline		[5,61,63–66]
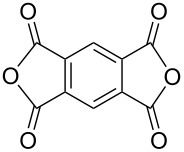	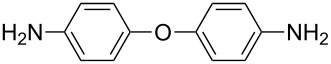		
furo[3,4-*f*][2]benzofuran-1,3,5,7-tetrone	1,4-diaminobenzene		[61,67]
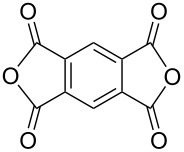	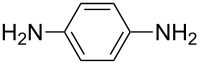		
furan-2,5-dione	1,4-diaminobenzene	furo[3,4-*f*][2]benzofuran-1,3,5,7-tetrone	[68]
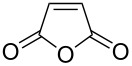	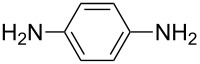	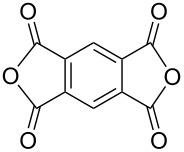	

Putkonen et al. [61] combined PMDA together with ethane-1,2-diamine (ED) and hexane-1,6-diamine to produce semi-aromatic polyimides. The highest growth rates achieved for these thin films were 3.9 Å per cycle for the former and 5.8 Å per cycle for the latter case, both at the deposition temperature of 160 °C. As the chain lengths for the ED and hexane-1,6-diamine containing polyimide units are 9.8 and 14.5 Å, respectively, the GPC values achieved are quite far from those expected for an ideal MLD growth [61]. In a later study Salmi et al. [62] also grew polyimide films from PMDA and hexane-1,6-diamine. The depositions were done at the temperature of 170 °C and a GPC of 5.6 Å per cycle was reported. The article concentrates on nanolaminates fabricated from Ta_2_O_5_ and the polyimide and will be discussed in more detail later in this review [62].

In all the other polyimides deposited by MLD the second precursor used has been aromatic. When 4-nitrobenzene-1,3-diamine was employed together with PMDA, no strong bonds formed between the two species, preventing the successful film growth [5].

The aromatic diamine precursor 4,4’-oxydianiline (ODA) has been used by several research groups. The highest growth rates reported were obtained by Yoshimura et al. [5]: In their experiments a GPC value as high as 6.7 Å per cycle on an unspecified substrate was achieved. In later experiments carried out by Putkonen et al. [61] the highest GPC value obtained was 4.9 Å per cycle on Si(100) and soda lime glass substrates at 160 °C. Yoshida et al. [63–64] employed Au-coated Si substrates, modified with 4-aminothiophenol to obtain NH_2_-group terminated surfaces. They conducted the experiments at 170 °C, achieving a GPC value of 2.0 Å per cycle. As the approximate length of the PMDA–ODA chain is 14.9 Å, none of the groups achieved GPC values close to full monolayer coverage. Yoshida et al. [63] also modified the films electrically by using a scanning probe microscope and reported significant increases in the conductivity of the films. The precursor combination, PMDA+ODA, was also employed by Haq et al. [65], who used reflection–absorption infrared spectroscopy to study the growth on Cu(110) surfaces as a function of temperature and coverage but did not discuss growth rates of the films. Also Miyamae et al. [66] deposited thin films by using PMDA and ODA, but as PMDA was introduced to the reactor so that the pulsing of ODA was kept on, the growth process was not necessarily of the real MLD type.

The combination, PMDA+1,4-diaminobenzene, was used to fabricate organic thin films by Putkonen et al. [61], and also by Bitzer and Richardson [67]. The former group achieved the highest GPC value of 1.4 Å per cycle, which is well below the calculated chain length of 14 Å, when using Si(100) and soda lime glass substrates at the deposition temperature of 160 °C. The latter group fabricated ultrathin films on Si(100)-2×1 at room temperature but no GPC value was reported. Bitzer and Richardson [68] later also deposited ultrathin films on Si(100)-2×1 first functionalized with maleic anhydride, followed by stepwise exposures of 1,4-diaminobenzene and PMDA at room temperature.

#### Polyimide–amides

Miyamae et al. [66] deposited polyimide–amide thin films by using PMDA and terephthaloyl dichloride as the first and third building blocks, whereas ODA or decane-1,10-diamine were used as the second one (Table 5). However, as with the PMDA–ODA films grown by the same group, also with the polyimide–amides the precursor pulses were overlapping and thus the process was not precisely of the MLD type.

**Table 5:** Precursors used to deposit polyimide–amide thin films.

precursor A	precursor B	precursor C	reference

furo[3,4-*f*][2]benzofuran-1,3,5,7-tetrone	4,4’-oxydianiline	terephthaloyl dichloride	[66]
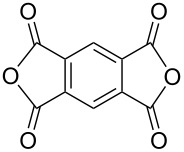	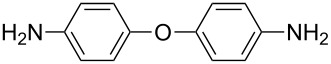	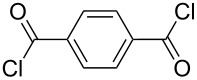	
furo[3,4-f][2]benzofuran-1,3,5,7-tetrone	decane-1,10-diamine	terephthaloyl dichloride	[66]
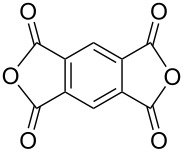	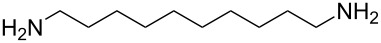	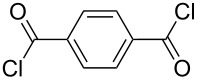	

#### Polyureas

Polyureas are tough elastomers with a high melting point. They are especially useful as protective coatings. The polymers form with a reaction between an isocyanate and an amine. Depending on the diisocyanate used to fabricate the polymer, polyureas can be divided to either aromatic or aliphatic systems. The aromatic polyureas are typically sensitive to light, changing color after exposure, whereas the aliphatic polyureas retain their color when treated similarly. Both types have been deposited by using MLD (Table 6).

**Table 6:** Precursors used to deposit polyurea thin films.

precursor A	precursor B	references

1,4-diisocyanatobenzene	ethane-1,2-diamine	[69–71]
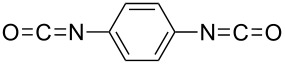	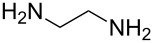	
1,4-diisocyanatobenzene	2,2’-(propane-2,2-diylbis(oxy))-diethanamine	[72]
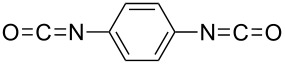	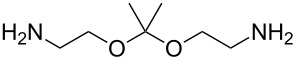	
1,4-diisocyanatobutane	ethane-1,2-diamine	[73]
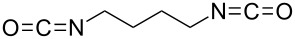	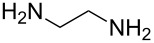	
1,4-diisocyanatobutane	*N*-(2-aminoethyl)ethane-1,2-diamine	[73]
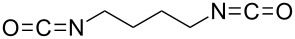	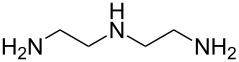	
1,4-diisocyanatobutane	*N*,*N*-bis(2-aminoethyl)ethane-1,2-diamine	[73]
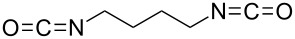	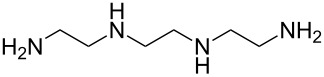	
1,4-diisocyanatobutane	tris(2-aminoethyl)amine	[73]
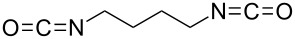	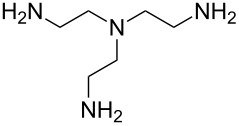	

1,4-diisocyanatobenzene (1,4-phenylene diisocyanate, PDIC) is the only aromatic diisocyanate so far used to fabricate polyureas by MLD. Kim et al. [69] employed ED as the second precursor, depositing the films on Ge(100)-2×1 at room temperature. The growth of the ultrathin films was investigated with multiple-internal-reflection Fourier-transform infrared spectroscopy, demonstrating the formation of urea linkages, although some imperfections in the growth, possibly due to double reactions of ED, were also observed. In a later study by Loscutoff et al. [70] the same precursors were used to fabricate thin films on Si(100), treated with 3-aminopropyltriethoxysilane before depositions to get amine-terminated surfaces. The deposition temperatures used ranged from 25 to 100 °C, and the GPC of the films decreased from 4.1 Å per cycle at 25 °C to 0.4 Å per cycle at 100 °C. A full monolayer coverage was not achieved as the highest GPC value was well below the PDIC+ED molecule length of 12.6 Å, and also below the somewhat lower GPC value of 6.5 Å per cycle expected based on the density functional theory calculations carried out by the group. The film growth was observed to be linear. The films were stable in ambient air and when annealed at least up to 250 °C [70]. Recently, Prasittichai et al. [71] achieved a GPC of 5.3 Å per cycle when growing PDIC+ED films at room temperature. In their study self-assembled monolayers fabricated by using octadecyltrichlorosilane were used to prevent the growth of the PDIC+ED polymer, enabling the growth of patterned 3D structures [71].

Zhou et al. [72] used 2,2’-(propane-2,2-diylbis(oxy))-diethanamine together with PDIC, and fabricated the films on Si(100) at room temperature. The growth rate of these films was ca. 6.5 Å per cycle, which is considerably less than the chain length of the expected molecule (18 Å). The study demonstrated that when treated with acid, the backbone of the formed film reacted from the acid-labile groups. When exposed to basic solution, the polymer films were stable. These experiments proved that MLD can be utilized to fabricate photoresist materials. To make the film a photoresist material, a treatment with triphenylsulfonium triflate, a photoacid generator, was required after the deposition.

Also aliphatic polyureas have been fabricated by MLD using only one diisocyanate, namely 1,4-diisocyanatobutane. ED, *N*-(2-aminoethyl)ethane-1,2-diamine, *N*,*N*-bis(2-aminoethyl)ethane-1,2-diamine and tris(2-aminoethyl)amine have been used as the second precursor, yielding cross-linked polyurea films with GPCs of 6.3, 6.7, 3.2, and 3.1 Å per cycle, respectively. The depositions were carried out at room temperature and Si(100) was used as the substrate. As the calculated chain lengths for the respective systems are 13.5, 17.2, 20.9, and 17.2 Å, the growth rates of the polymers were considerably less than a full monolayer per cycle [73].

#### Polyurethanes

As with polyureas, the reaction in which polyurethane formation takes place involves an isocyanate, but instead of an amine, an alcohol is required for the urethane linkage. In the MLD-grown polyurethanes the isocyanate has been PDIC, whereas but-2-yne-1,4-diol and terephthalic acid bis(2-hydroxyethyl) ester has been used as the second precursor in experiments done by Lee et al. [74] (Table 7). As the aim of the group was to make templates for the synthesis of zeolites, the growth process of the films is not discussed in detail. However, from the thickness evaluations for the system PDIC+but-2-yne-1,4-diol, which vary from 9.5 to 6.1 Å per cycle, it can be seen that the growth is not linear.

**Table 7:** Precursors used to deposit polyurethane thin films.

precursor A	precursor B	reference

1,4-diisocyanatobenzene	but-2-yne-1,4-diol	[74]
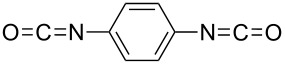	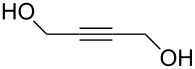	
1,4-diisocyanatobenzene	terephthalic acid bis(2-hydroxyethyl) ester	[74]
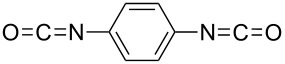	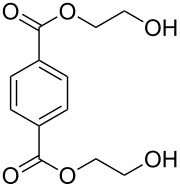	

#### Polythiourea

The only MLD-grown polythiourea was fabricated by using 1,4-diisothiocyanatobenzene and ED as the precursors (Table 8). The deposition temperature in these experiments was kept at 50 °C and the films were grown on silicon and silica nanoparticles. The obtained growth rates were 1.9 and 2.8 Å per cycle, respectively. These values are modest when comparing to the calculated chain length of 13.8 Å [75].

**Table 8:** Precursors used to deposit polythiourea thin films.

precursor A	precursor B	reference

1,4-diisothiocyanatobenzene	ethane-1,2-diamine	[75]
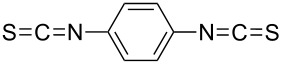	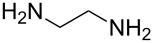	

#### Polyesters

Polyesters are commonly made with reactions between various acids and alcohols. The only MLD polyester reported is polyethylene terephthalate (PET), which is an industrially widely used polymer. The precursors employed for the fabrication of PET films were terephthaloyl dichloride, which is an acid chloride, and ethane-1,2-diol (ethylene glycol, EG), a diol (Table 9). The deposition temperature range investigated was 145–175 °C. The films were grown on Si(100) substrates, cleaned ultrasonically in ethanol and water. The depositions were carried out on both cleaned substrates and on substrates that were further functionalized with amine groups using (3-aminopropyl)triethoxysilane. The growth rates were significantly better with the functionalized substrates. The highest growth rate, 3.3 Å per cycle, was obtained at 145 °C. The length of a PET molecule is 11 Å, so only partial layer coverage was achieved, though [76].

**Table 9:** Precursors used to deposit polyester thin films.

precursor A	precursor B	reference

terephthaloyl dichloride	ethane-1,2-diol	[76]
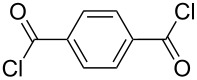	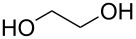	

#### Polyimines

Terephthalaldehyde and 1,4-diaminobenzene have been used to form polyimine thin films (Table 10) at room temperature [9,77–78]. The growth process was investigated by using Au-coated glass substrates with a self-assembled monolayer of 11-amino-1-undecanethiol. According to the investigations by Yoshimura et al. [78] the polymer wires grew in upward directions. Later experiments showed that that the first six cycles yielded a GPC value of 10 Å per cycle, after which the growth rate started to decline [77]. Different combinations of terephthalaldehyde, 1,4-diaminobenzene and ethanedihydrazide have been used to fabricate quantum dots of varying lengths: These thin films showed promise for sensitization in photovoltaic devices [79–80].

**Table 10:** Precursors used to deposit polyimine thin films.

precursor A	precursor B	precursor C	references

terephthalaldehyde	1,4-diaminobenzene		[9,77–78]
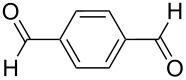	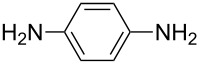		
terephthalaldehyde	1,4-diaminobenzene	ethanedihydrazide	[79–80]
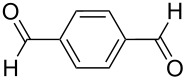	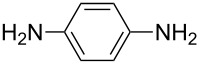	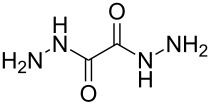	

### Hybrid inorganic–organic thin films

Since the first publications featuring inorganic–organic hybrid thin films, the number of articles relating to this type of films has been rapidly increasing. In the following subchapters the inorganic–organic thin films deposited by using the combined ALD and MLD technique are presented. The different thin films produced are divided by the organic precursor employed. The subchapters “Alcohols and phenols”, “Acids” and “Amines” contain all the different hybrid films made by using an organic precursor with hydroxyl, carboxyl or nitrogen atom functionality, respectively. It should be noted that some of precursors under “Alcohols and phenols” have also another functional group, such as 4-aminophenol with both –OH and –NH_2_ groups. (2*S*)-2-Aminopentanedioic acid, an amino acid with carboxylic acid functionality, is considered under “Acids”. “Other organic precursors” consist of all the organic precursors that do not fall clearly into the categories mentioned above. The thin films based on 7-octenyltrichlorosilane are presented in their own subchapter at the end of this chapter.

#### Alcohols and phenols

**Figure 6:**
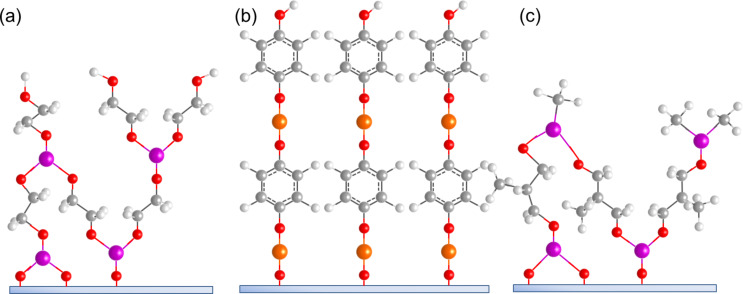
Schematic illustration of ALD/MLD inorganic–organic hybrid thin films deposited by using (a) TMA with ethane-1,2-diol, (b) DEZ with benzene-1,4-diol, and (c) TMA with oxiran-2-ylmethanol.

Most of the inorganic–organic hybrid thin films reported so far have been deposited by using alcohols or phenols as the organic precursor (Table 11). When metal precursors are combined with alcohols or phenols, the resultant material is often called “metalcone” [27]. Most of the research has been focused on the aluminum-based alucones [12–13,15–16,38–39,81–97], but also zincones [13,33,38–39,46,51–52,56,94–95,98–103], titanicones [53,55,95,104–105], and zircones [54,87] have been studied. The schematic structure of ideally growing TMA+ethane-1,2-diol, DEZ+benzene-1,4-diol and TMA+oxiran-2-ylmethanol hybrid films are shown in Figure 6 as examples of these types of films.

**Table 11:** Alcohol and phenol precursors used to deposit inorganic–organic hybrid thin films.

organic precursor	inorganic precursor	references

ethane-1,2-diol	Al(CH_3_)_3_	[12,15–16,81–89,91–92,95–96]
	Zn(C_2_H_5_)_2_	[51–52,98,101]
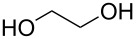	TiCl_4_	[53,104–105]
	Zr(C_4_H_9_O)_4_	[54,87]

propane-1,2,3-triol	Al(CH_3_)_3_	[95]
	Zn(C_2_H_5_)_2_	[95]
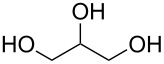	TiCl_4_	[53,95,105]

hexa-2,4-diyne-1,6-diol	Zn(C_2_H_5_)_2_	[46,56]
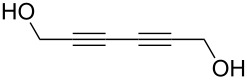	TiCl_4_	[55]

benzene-1,4-diol	Al(CH_3_)_3_	[13,94]
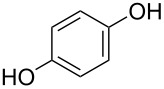	Zn(C_2_H_5_)_2_	[33,95,99–103]

benzene-1,3,5-triol	Al(CH_3_)_3_	[13]
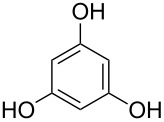		

oxiran-2-ylmethanol	Al(CH_3_)_3_	[38–39,90,93,97]
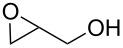	Zn(C_2_H_5_)_2_	[38–39]

4-aminophenol	Zn(C_2_H_5_)_2_	[36,103,106]
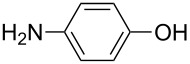	TiCl_4_	[37]

8-quinolinol	Al(CH_3_)_3_	[13,35]
	Zn(C_2_H_5_)_2_	[13,35]
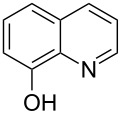	TiCl_4_	[13,35]

tris(2-hydroxyethyl)amine	Al(CH_3_)_3_	[107]
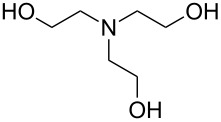		

2-aminoethanol + furan-2,5-dione	Al(CH_3_)_3_	[16,48,84,108–109]
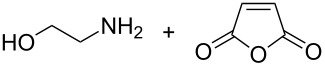		

2-aminoethanol + propanedioyl dichloride	TiCl_4_	[49]
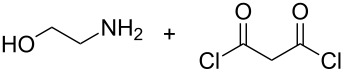		

Ethane-1,2-diol (ethylene glycol, EG) is the simplest diol used to fabricate inorganic–organic hybrid materials by ALD/MLD. The use of EG together with TMA was first reported by Dameron et al. [12]. The thin films were grown in the temperature range of 85–175 °C. The GPC value for the films varied from 4.0 to 0.4 Å per cycle, decreasing with increasing deposition temperature. The TMA+EG hybrids were unstable in ambient conditions, but capping with SiO_2_ improved the stability. Mechanical studies of the TMA+EG hybrids showed that the material is brittle, with a toughness of about 0.17 MPa·m^0.5^ [81]. The brittleness was also observed with nanointendation measurements, giving an elastic modulus of about 37 GPa and a Berkovich hardness of about 0.47 GPa [84]. However, when the TMA+EG hybrid with an additional H_2_O pulse was employed as an interlayer between ALD-grown Al_2_O_3_ and a Teflon substrate, it was noticed that the stress caused by the difference in the coefficient of thermal expansion between the coating and the substrate was significantly reduced, preventing the cracking of the Al_2_O_3_ coating [96].

The first zincones were fabricated by combining EG with DEZ. This type of hybrid was reported by Peng et al. [51] and Yoon et al. [52]. The former group deposited the thin films in the temperature range of 100–170 °C. The maximum GPC value, about 0.57 Å per cycle, was achieved for the film deposited at 120 °C, while the lowest GPC, about 0.39 Å per cycle, was observed for the films deposited at 165 °C. However, the DEZ+EG hybrids are unstable in ambient air and the thickness measurements were carried out on reacted films with a reduced thickness. The films by Yoon et al. [52] were deposited at temperatures between 90 and 170 °C. Unlike with the depositions by Peng et al. [51], the thickness of the films decreased with increasing temperature. A growth rate of 0.7 Å per cycle was measured for films deposited at 130 °C. Both groups reported that although the DEZ+EG hybrid reacts with water in ambient air, the films remained stable after an initial quick reaction. Recently Liu et al. [101] grew DEZ+EG hybrid films at 150 °C. They observed that the GPC varied strongly depending on the number of deposition cycles, from 0.39 to 0.86 Å per cycle for 500 and 2000 cycles, respectively. This phenomenon was speculated to be due to the double reactions occurring during the growth. Also the GPC is far below the length of a Zn–EG unit, which was estimated to be around 6.9 Å. Thermal conductivity of the DEZ+EG hybrid was measured to be around 0.22–0.23 W/(m·K), with little variation in the values when thicker samples were analyzed. The volumetric heat capacity was about 2.5 J/(cm^3^·K) [101].

Ethylene glycol has also been used with TiCl_4_ to deposit titanicone films [53,104–105]. Deposition temperatures for the TiCl_4_+EG films varied from 90 to 135 °C. The GPC first decreased gradually from 4.6 Å per cycle at 90 °C to 4.1 Å per cycle at 125 °C, after which there was a larger drop to 1.5 Å per cycle at 135 °C. The TiCl_4_+EG hybrid thin films were unstable: The thickness diminished by 15% over five days and after 25 days the total reduction was 20%. The elastic modulus and hardness values measured by using nanointendation were extremely low, i.e., about 8 GPa and about 0.25 GPa, respectively [53].

Zircone thin films have been fabricated by using EG together with zirconium *tert*-butoxide (ZTB) [54,87]. The depositions were carried out in the temperature range of 105–195 °C. The GPC decreased with increasing deposition temperature, from 1.6 to 0.3 Å per cycle. The films were stable in ambient air: A decrease in thickness of only ca. 3% was observed after exposure in ambient air for one month [54].

Titanicone films have been also made by using propane-1,2,3-triol (glycerol, GL) together with TiCl_4_. The deposition temperature regime used for these films was between 130 and 210 °C. The GPC value at 130 °C was 2.8 Å per cycle and dropped with increasing temperature gradually to 2.1 Å per cycle at 210 °C. A small increase of film thickness was observed when thin films were exposed to air. The elastic modulus and hardness were both higher for the GL titanicones than those based on EG, with values of about 30.6 GPa and about 2.6 GPa, respectively. The films were also more thermally stable than the EG-based counterparts, probably due to crosslinking in the structure [53].

Hexa-2,4-diyne-1,6-diol (HDD) has been combined together with DEZ and TiCl_4_ to form cross-linked polydiacetylene structures. These films were polymerized by using UV irradiation after the DEZ/TiCl_4_ and HDD pulses. Deposition temperatures of 100–150 °C, and 100 °C were used for the Zn- and Ti-containing hybrid films, respectively. The calculated ideal layer thickness for both types of hybrids was approximately 6 Å. The GPC of the DEZ+HDD films was close to the ideal, i.e., 5.2 Å per cycle, whereas the measured GPC for the TiCl_4_+HDD system reached full mono layer coverage, i.e., 6 Å per cycle. Investigation of the electrical properties of the DEZ+HDD thin films revealed the films had an excellent field effect mobility (>1.3 cm^2^·V^−1^·s). When TEM was used to observe structures obtained with 50 cycles of TiO_2_ and one cycle of TiCl_4_+HDD, individual nanolayers from each material could be seen [55–56].

Benzene-1,4-diol (hydroquinone, HQ), a phenol with two –OH groups in a para position, and TMA has been used to deposit films in the temperature range of 150–400 °C. The GPC was quite constant regardless of the deposition temperature used, around 3.5 Å per cycle. When the films were stored in ambient air for one week, a decrease of 25% in film thickness was measured [13]. Zn-containing HQ films have also been fabricated with DEZ. The DEZ+HQ films were grown at temperatures between 130 and 170 °C, yielding GPC of 1.6 Å per cycle at 150 °C. The stability of these films was not discussed [33]. In another study, a GPC of 2.7 Å per cycle at the deposition temperature of 150 °C was obtained. Although considerably higher than when comparing to GPC obtained earlier, it is still far from the ideal growth, which was estimated to be around 8.4 Å per cycle. Thermal conductivity of the DEZ+HQ films was considerable higher than those of the DEZ+EG films, i.e., about 0.32–0.38 W/(m·K): The difference was attributed to a more vertical growth in case of the hybrid using the aromatic precursor when compared to the one using the linear precursor. Also the volumetric heat capacity was higher with the DEZ+HQ than with DEZ+EG, i.e., about 3.1 J/(cm^3^·K) [101].

Benzene-1,3,5-triol, an aromatic compound with three –OH groups, has been used to fabricate hybrid thin films together with TMA. The depositions were carried out in temperature range of 175–400 °C. The GPC was about 5 Å per cycle regardless of the used deposition temperature. When stored in ambient air for one week, an increase of 9% in film thickness was measured [13].

There have been several studies featuring inorganic–organic hybrid materials deposited by using TMA and oxiran-2-ylmethanol (glycidol, GLY) [38–39,90,93,97]. The GLY molecule is heterobifunctional with both hydroxy and epoxy functionalities. Heterobifunctionality is believed to reduce the number of double reactions. During the deposition an epoxy ring-opening reaction takes place: The reaction is strongly dependent on the use of strong Lewis acids such as TMA. The first detailed studies of the growth of TMA+GLY thin films were published by Gong et al. [38] and Lee et al. [39] The temperature regimes used by the two groups were 90–150 °C and 100–175 °C, respectively. The GPC values reported varied greatly. Gong et al. [38] reported GPC values which decreased with increasing deposition temperature, the values varying from 24 (which is considerably higher than the calculated chain length) to 6 Å per cycle, whereas Lee et al. [39] reported a growth rate of only 1.3 Å per cycle at 125 °C. Both groups reported that the TMA+GLY thin film growth is sensitive to reaction conditions. As the paper by Lee et al. [39] was the one published a little later, they speculated that the difference in their GPC values when compared to those reported by Gong et al. [38] could be due to CVD-type growth regime at lower deposition temperatures and with short purging times. Gong et al. [38] observed that the TMA+GLY films were relatively stable in ambient air: A 168 hour exposure resulted in the absorption of some OH^−^ according to FTIR data, but no change in thickness was detected. Both groups performed experiments also with DEZ+GLY systems, but no decent growth was observed for these films. It was concluded that DEZ as a weaker Lewis acid is not able to sufficiently catalyze the reaction required for the film growth to proceed [38–39].

The 4-aminophenol (AP) molecule is heterobifunctional consisting of a benzene ring with both –OH and –NH_2_ groups. It has been investigated together with the inorganic precursor DEZ. The DEZ+AP hybrid thin film depositions were carried out at temperatures between 140 and 330 °C. Rather constant GPC of about 1.1 Å per cycle was obtained at deposition temperatures of 140–200 °C, after which the GPC started to decrease with increasing temperature. The resultant films were stable when stored in ambient air when the humidity level was low [36]. Recently AP was used together with TiCl_4_, deposited at temperatures between 120 and 220 °C. The obtained GPC, which was 10–11 Å per cycle in the deposition temperature range of 140–160 °C, was close to the value calculated from the bond lengths, 9.1 Å per cycle. The films were relatively stable: Less than 10% decrease in film thickness was observed when stored in ambient air for 800 h [37].

Aromatic 8-quinolinol has been used together with TMA, DEZ and TiCl_4_ [13,35]. The deposition temperature ranged from 85 to 200 °C. The maximum growth rates were about 4, 6.5 and 7.5 Å per cycle for the Al-, Zn-, and Ti-containing hybrids, respectively, and were obtained at the lowest deposition temperature used for each system. The growth rate gradually decreased with increasing temperature for all processes and no growth was observed in the films deposited at 200 °C. Significant photoluminescent activity was observed for the Al- and Zn-based hybrids whereas in the Ti-containing films a detectable but only small activity was perceived [35].

Tris(2-hydroxyethyl)amine, a tertiary amine, has been combined together with TMA to form hybrid thin films. The depositions were performed at 150 °C. The GPC was 1.3 Å per cycle and the films were unstable in ambient air [107].

The use of more than two precursors can mitigate the probability of double reactions. Yoon et al. [48] were the first to make inorganic–organic hybrid thin films by using three different precursors, namely TMA, 2-aminoethanol and furan-2,5-dione. From the two organic precursors, 2-aminoethanol is heterobifunctional while furan-2,5-dione is a ring-opening reactant. The depositions were done in the temperature range of 90–170 °C. The GPC was 24 Å per cycle at 90 °C and 4.0 Å per cycle at 170 °C. The films were not stable: It was observed that the films react with water mostly within the first 10 min of exposure to ambient air [48]. In later studies it was deduced that the growth process of the films is governed by the TMA diffusion into and out of the forming film, making the growth process strongly dependent on the TMA dose and purge times used. Also experiments carried out at 130 °C showed that the self-limiting growth could be only observed when the films remained thin [108]. According to nanointendation measurements conducted for the TMA+2-aminoethanol+furan-2,5-dione films deposited at 90 °C, the elastic modulus and Berkovich hardness were about 13 GPa and about 0.27 GPa, respectively [84].

Later, Chen et al. [49] also utilized three different precursors to fabricate hybrid thin films, i.e., TiCl_4_, 2-aminoethanol and propanedioyl dichloride. Unlike with the TMA+2-aminoethanol+furan-2,5-dione system, the growth sequence of these films did not consist of repeated introduction of the precursors in three stages, but in four: One full deposition cycle consisted of supplying TiCl_4_, followed by 2-aminoethanol, then propanedioyl dichloride and finally a repeated pulsing of 2-aminoethanol. The heterobifunctionality of the 2-aminoethanol molecule was expected to improve reaction selectivity of the process. A GPC value of 6 Å per cycle was achieved at deposition temperature of 100 °C when grown on carbon nanocoils.

#### Acids

**Figure 7:**
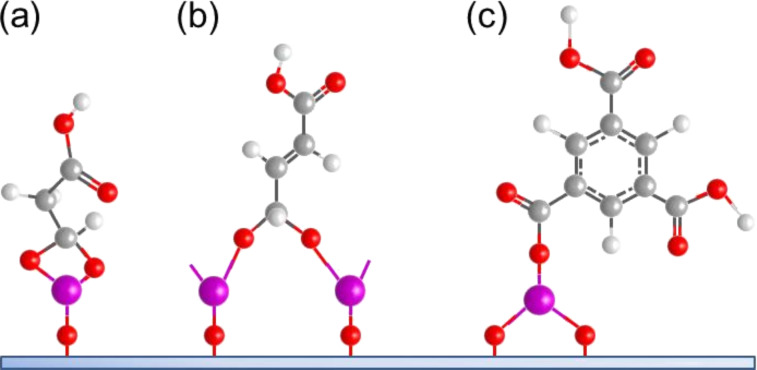
Schematic illustration of ALD/MLD inorganic–organic hybrid thin films deposited using TMA together with (a) propanedioic acid (bidentate complex), (b) (*E*)-butenedioic acid (bridging complex), and (c) benzene-1,3,5-tricarboxylic acid (unbidentate complex).

Although the deposition of inorganic–organic hybrid thin films by using carboxylic acids as organic precursors was first mentioned by Nilsen et al. [13] already in 2008 (Table 12), the later articles published by the same group discuss the topic in greater detail [32,57,110], separately for saturated [57] and unsaturated [110] linear acids, as well as aromatic acids [32], when combined with TMA. The saturated linear acids were observed to form mostly bidentate complexes, and the unsaturated linear acids formed either bidentate or bridging complexes, whereas for the aromatic acids the complex type varied depending on the acid. Only one carboxylic acid has been used in combination with an inorganic precursor different from TMA, namely benzene-1,4-dicarboxylic acid with zinc acetate [111]. Representatives for the possible film structures are depicted in Figure 7.

**Table 12:** Carboxylic acid precursors used to deposit inorganic–organic hybrid thin films.

organic precursor	inorganic precursor	references

ethanedioic acid	Al(CH_3_)_3_	[13,57]
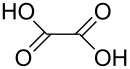		
propanedioic acid	Al(CH_3_)_3_	[13,57]
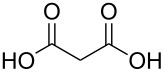		
butanedioic acid	Al(CH_3_)_3_	[57]
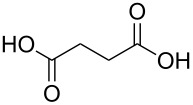		
pentanedioic acid	Al(CH_3_)_3_	[13,57,112]
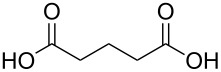		
propane-1,2,3-tricarboxylic acid	Al(CH_3_)_3_	[112]
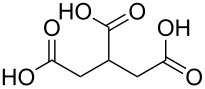		
heptanedioic acid	Al(CH_3_)_3_	[13,57]
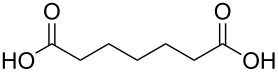		
octanedioic acid	Al(CH_3_)_3_	[13,57]
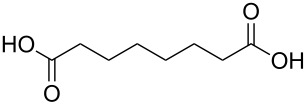		
decanedioic acid	Al(CH_3_)_3_	[13,57]
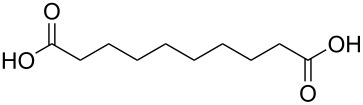		
(*E*)-butenedioic acid	Al(CH_3_)_3_	[13,110]
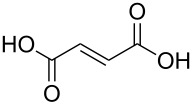		
(*Z*)-butenedioic acid	Al(CH_3_)_3_	[13,110]
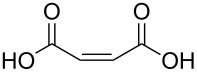		
(1*E*)-prop-1-ene-1,2,3-tricarboxylic acid	Al(CH_3_)_3_	[110]
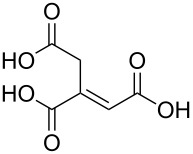		
(2*E*,4*E*)-hexa-2,4-dienedioic acid	Al(CH_3_)_3_	[13,110]
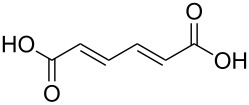		
benzoic acid	Al(CH_3_)_3_	[32]
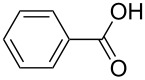		
benzene-1,2-dicarboxylic acid	Al(CH_3_)_3_	[13,32]
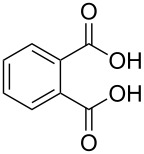		
benzene-1,3-dicarboxylic acid	Al(CH_3_)_3_	[13,32]
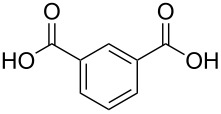		
benzene-1,4-dicarboxylic acid		
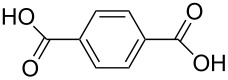	Al(CH_3_)_3_ Zn(CH_3_CO_2_)_2_	[13,32] [111]
benzene-1,3,5-tricarboxylic acid		
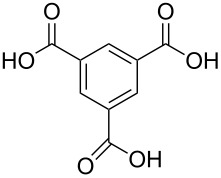	Al(CH_3_)_3_	[13,32]
benzene-1,2,4,5-tetracarboxcylic acid	Al(CH_3_)_3_	[13,32]
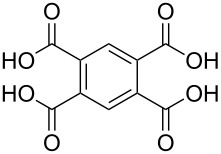		
(2*S*)-2-aminopentanedioic acid	Al(CH_3_)_3_	[112]
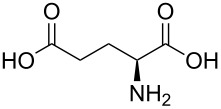		

**Figure 8:**
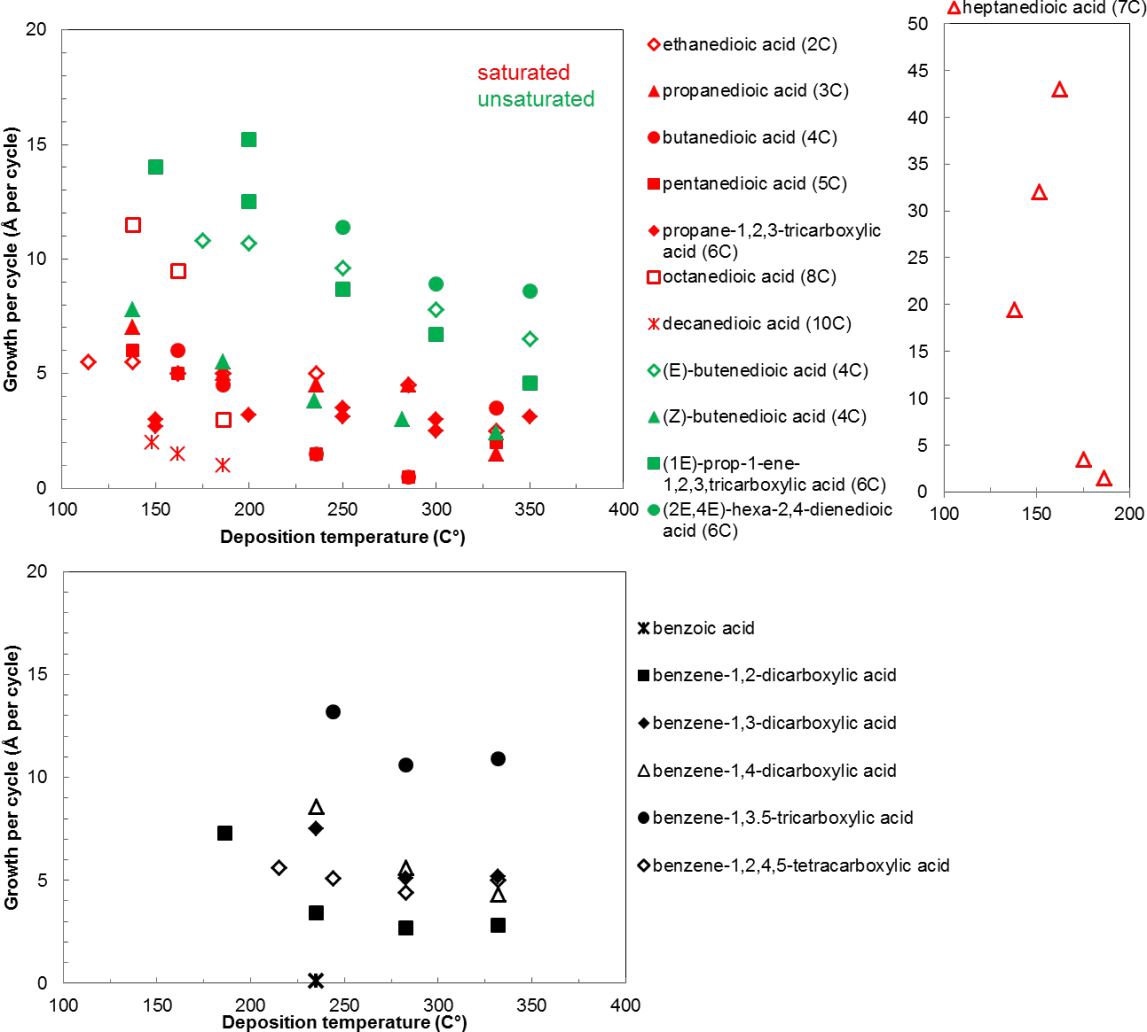
Growth per cycle values for inorganic–organic hybrid films deposited by using TMA with different carboxylic acids [32,57,110,112].

In the works reported for the combination, TMA+saturated linear acid, the chain length of the acid varies from two carbon atoms for the ethanedioic acid to ten carbon atoms for the decanedioic acid. Accordingly, the GPC values for the different systems also vary greatly (Figure 8), from 43 Å per cycle achieved with the heptanedioic acid with seven carbon atoms to about 1 Å per cycle for the decanedioic acid. From Figure 8, all the other systems but the one using heptanedioic acid and propane-1,2,3-tricarboxylic acid show a decrease in GPC with increasing deposition temperature. For the heptanedioic acid the GPC is highly dependent on the deposition temperature, and the maximum value attained at 162 °C is even four times higher than the chain length of the acid precursor, a detail which could not be explained by the authors. The GPC of propane-1,2,3-tricarboxylic acid remained constant in the temperature range investigated. From Figure 8, the maximum GPC values for the acids containing 2–5 carbon atoms were of the same order of magnitude, whereas higher values were obtained for the acid systems with seven and eight carbon atoms and lower values for the decanedioic acid with ten carbons [57,112].

From Figure 8, when disregarding the heptanedioic acid+TMA hybrid, the GPCs for the unsaturated acids employed were higher when compared to those of saturated acids. The maximum GPCs varied between 7.8 and about 15 Å per cycle obtained for (*Z*)-butenedioic acid and (1*E*)-prop-1-ene,1,2,3-tricarboxylic acid systems. For all systems, the GPC decreased with increasing deposition temperature [110,112].

The aromatic carboxylic acids used to fabricate hybrid films together with TMA vary from each other regarding the number of carboxyl groups attached to the aromatic ring and how the groups are positioned. Benzoic acid has only one carboxyl group, which may explain why no decent growth for these films were observed. From dicarboxylic acids all the possible group positions were investigated, i.e., benzene-1,2-dicarboxylic acid, benzene-1,3-dicarboxylic acid and benzene-1,4-dicarboxylic acid. The maximum GPC value for all these three acids, as seen from Figure 8, was in the range of 8 ± 1 Å per cycle. The aromatic carboxylic acid containing three carboxylic groups, benzene-1,3,5-tricarboxylic acid, produced the highest GPC values from the aromatic acids: The maximum GPC was as high as 13.4 Å per cycle. The maximum GPC of benzoic-1,2,4,5-tetracarboxylic acid was considerably lower, 5.6 Å per cycle. As can be seen from Figure 8, for all aromatic acid systems the GPC decreases with increasing deposition temperature [32].

All the carboxylic acid+TMA hybrid films were stable: No changes were observed for the saturated and unsaturated acid systems when stored in ambient air for one year, whereas the stability of aromatic carboxylic acids was observed for one week [32,57,110,112].

Salmi et al. [111] used benzene-1,4-dicarboxylic acid together with zinc acetate to deposit metal-organic framework (MOF) thin films. The films were deposited at 225–350 °C, and the GPC decreased with increasing deposition temperature, the highest value being 6.5 Å per cycle. No growth was observed when the deposition was carried out at 350 °C. The as-deposited films were amorphous, but films deposited below 300 °C crystallized when kept at 60% humidity at room temperature. According to the time-of-flight elastic recoil detection analysis and FTIR experiments conducted for the crystallized films, the composition was close to that of MOF-5.

Klepper et al. [112] used (2*S*)-2-aminopentanedioic acid, an amino acid with a carboxylic acid functional group, together with TMA. The films were grown in the temperature range of 200–350 °C. The GPC values decreased with increasing deposition temperature. The highest GPC obtained was 20 Å per cycle, which is considerably higher than the estimated chain length, which was 10 Å. However, according to the XRD measurements, there was a peak around 4.5° in the θ–2θ pattern, which corresponds to a *d* value of 19.9 Å, indicating an Al–Al sheet distance, which is close to the maximum GPC. The group concluded that a sheet-like structure with an interplanary sheet distance of 20 Å forms during the deposition of the film. Also these films were stable: No changes were observed visually or in XRR and FTIR measurements.

#### Amines

Two diamines, 1,4-diaminobenzene and ODA, have been used to fabricate inorganic–organic hybrid thin films by ALD/MLD (Table 13). The 1,4-diaminobenzene, i.e., an amine equivalent of HQ, was used together with TMA. The depositions were carried out in the temperature range of 200–400 °C. The GPC decreased with increasing deposition temperature, varying between about 1 and about 2 Å per cycle. The obtained films were unstable: An increase of ca. 30% in film thickness was observed when the films were kept in ambient air for two weeks [34].

**Table 13:** Amine precursors used to deposit inorganic–organic hybrid thin films.

organic precursor	inorganic precursor	references

1,4-diaminobenzene	Al(CH_3_)_3_	[34]
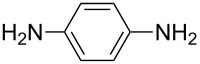		
4,4’-oxydianiline	TiCl_4_	[14,103,113]
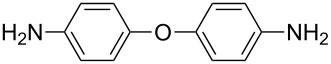		

The amine precursor ODA has been used together with TiCl_4_ to deposit thin films at deposition temperatures varying from 160 to 490 °C. GPC of these films increased with increasing deposition temperature, from 0.3 Å per cycle to 1.1 Å per cycle with an ODA-pulse length of 3 s. The TiCl_4_ films were stable in ambient air when deposited at 250 °C and above. Wet-etching testing revealed that when treated with toluene, acetone, methanol, 1 M acetic acid, or water, no significant change in film thickness was observed [14]. However, as the ODA-pulse length of 3 s was not enough for the fully-saturated growth, in a later study TiCl_4_+ODA films with longer ODA pulse lengths were deposited. It was observed that an ODA pulse length of 12–16 s led to saturated growth. Depositions at temperatures from 160 to 310 °C were carried out with an ODA pulse length of 14 s. The GPC of these films also increased with increasing temperature, from 0.8 Å per cycle at 160 °C to 1.6 Å per cycle at 310 °C [113].

#### Other organic precursors

Only three organic precursors have been used that cannot be included in the alcohol/phenol, acid or amine categories, namely ethanetetracarbonitrile, which is, like the name indicates, a nitrile, 2-oxepanone, a cyclic ester, and 1,2-bis[(diamethylamino)dimethylsilyl]ethane, which is a hybrid material in itself (Table 14).

**Table 14:** Various unclassified organic precursors used to deposit inorganic–organic hybrid thin films.

organic precursor	inorganic/counter precursor	reference

ethanetetracarbonitrile	V(CO)_6_	[114]
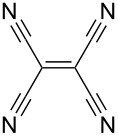		
2-oxepanone	Al(CH_3_)_3_	[47]
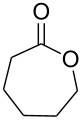		
1,2-bis[(dimethylamino)dimethylsilyl]ethane	O_3_	[115]
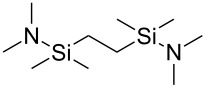		

Ethanetetracarbonitrile has been used together with vanadium hexacarbonyl V(CO)_6_ to produce the only vanadium-containing hybrid thin film deposited until now. The depositions were carried out at room temperature. A GPC of about 9.8 Å per cycle was achieved. The film was a room-temperature magnet, with a large coercive field (ca. 80 Oe at 5 and 300 K). According to the experiments, the hybrid has a high *T*_C_ and/or high thermal stability when compared to corresponding CVD-made films. It was concluded that the material holds promise for next-generation spin-related applications [114].

Gong et al. [47] used 2-oxepanone and TMA to make hybrid thin films in which the TMA as a Lewis acid catalyzes a ring-opening reaction. The films were deposited at 60–120 °C. The GPC value decreased with increasing temperature being 0.75 Å per cycle at 60 °C GPC but only 0.08 Å per cycle at 120 °C. The films were stable; no change in film thickness was observed when stored in ambient air for 30 days.

The hybrid film fabricated by Zhou and Bent [115] differs in some aspects from the other hybrids made up till now. It is the only carbosiloxane film made so far and the precursor used is an inorganic–organic hybrid material in itself, namely 1,2-bis[(dimethylamino)dimethylsilyl]ethane. Both O_3_ and H_2_O were tried as the second precursor, but H_2_O did not work as well as O_3_. It was speculated that double reactions hinder the growth when using H_2_O, whereas when O_3_ was used new surface groups were generated, improving the overall growth process. GPC of 0.2 Å per cycle was achieved at the deposition temperature of 110 °C for the 1,2-bis[(dimethylamino)dimethylsilyl]ethane+O_3_ system. The films were extremely stable, withstanding well wet etching treatments of tetramethylammonium hydroxide, HCl and acetone as well as vacuum annealing: When annealed at 300 °C, no change in thickness was observed, 400 °C resulted in a thickness change of 6%, and after annealing at 600 °C, a thickness loss of 13 % was measured.

#### 7-octenyltrichlorosilane based thin films

**Figure 9:**
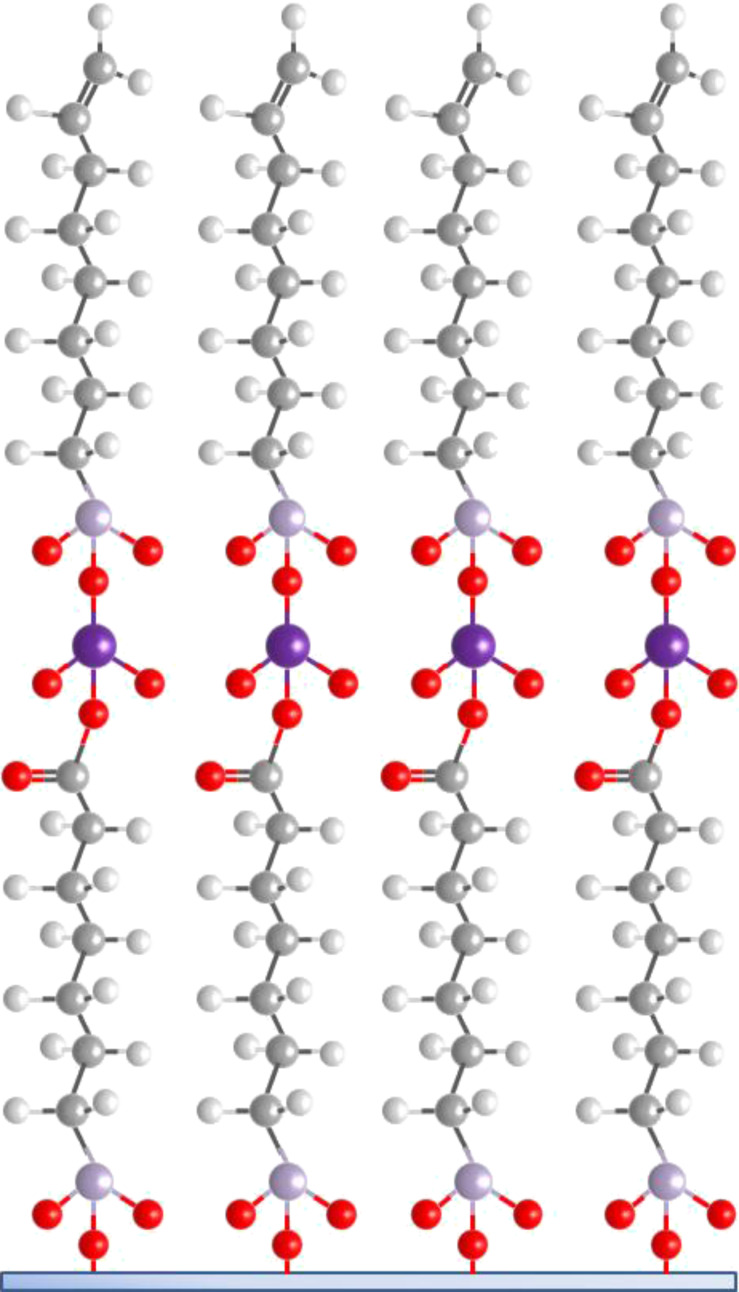
Schematic illustration of an ALD/MLD inorganic–organic hybrid thin film deposited using 7-octenyltrichlorosilane and Ti(OCH(CH_3_)_2_)_4_.

ALD/MLD has been used to deposit several different inorganic–organic hybrid films featuring 7-octenyltrichlorosilane (7-OTS) as one of the precursors (Table 15). The first article where 7-OTS was employed was published already in 2007 [10]. However, 7-OTS in itself cannot be said to be a fully organic material as it contains silicon. Also 7-OTS was pulsed to the reactor together with water as a catalyst, not alone as in a conventional ALD/MLD process. The key stages for all these 7-OTS hybrids during one cycle were the same: First 7-OTS and water were introduced, followed by an ozone treatment. The third stage consisted of metal precursor connection to the forming chain, followed by reaction with water. Schematic presentation of the forming chain is shown in Figure 9. The metal precursors used to make these type of hybrid thin films include Ti(OCH(CH_3_)_2_)_4_ [10,40], TMA [46], Zr(C_4_H_9_O)_4_ [43] and DEZ [44]. For all but TMA thin films the GPC was around 10–11 Å per cycle. The maximum growth rate for the 7-OTS+TMA film was only 6 Å per cycle. There are some contradictions regarding the reported growth rates for a full monolayer covering. According to the earlier articles an ideal monolayer would have a length of 12 Å [10,41] whereas a more recent one states that the full monolayer coverage would lead to a GPC value of 25 Å per cycle [40].

**Table 15:** Inorganic–organic hybrid thin film systems based on 7-octenyltrichlorosilane as a precursor.

precursors of organic layer		precursors of inorganic layer(s)			references

7-octenyltrichlorosilane	H_2_O	O_3_	Ti(OCH(CH_3_)_2_)_4_	H_2_O	10,40
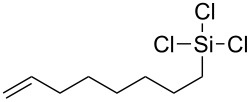					
7-octenyltrichlorosilane	H_2_O	O_3_	Ti(OCH(CH_3_)_2_)_4_ Al(CH_3_)_3_	H_2_O	41,42
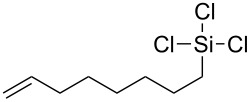					
7-octenyltrichlorosilane	H_2_O	O_3_	Zr(C_4_H_9_O)_4_	H_2_O	43
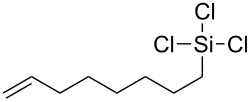					
7-octenyltrichlorosilane	H_2_O	O_3_	Zn(C_2_H_5_)_2_	H_2_O	44
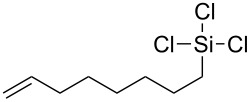					
7-octenyltrichlorosilane	H_2_O	O_3_	Al(CH_3_)_3_	H_2_O	45,46
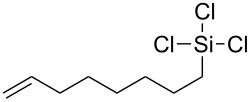					

The 7-OTS+Ti(OCH(CH_3_)_2_)_4_ containing films have been grown in a temperature range of 150–200 °C. When annealed, these films were stable up to 450 °C. When the electrical properties of the films were investigated, the experiments showed that the current density decreased with increased film thickness. When the films were less than 6.7 nm thick, direct tunneling was observed while the 99 nm thick films had a good gate leakage resistance. The dielectric constant for the films was around 17 at 1 MHz. It was concluded that the system 7-OTS+Ti(OCH(CH_3_)_2_)_4_ is a good candidate for high-quality gate-insulating films on flexible substrates [10]. In later experiments rapid water permeation speed was observed for this type of film [40].

Although the first article mentioning the use of 7-OTS together with TMA was published already in 2008 [41], a more thorough investigation of the growth process was carried out only recently [45]. The GPC decreased with increasing temperature, from about 6 Å per cycle at 100 °C to 2.5 Å per cycle at 200 °C [45].

The Zr- and Zn-containing films were grown at 170 and 150 °C, respectively. These studies focus more on the nanolaminates fabricated with oxides and will be discussed in more detail later [43–44].

### Processes with no growth details

In this chapter ALD/MLD works about only thin films that provide no precise details regarding the experiments are shortly summarized. It should be noted that the organic precursors used in the growth processes are not included in any of the tables.

The only Fe-containing hybrid films reported so far were grown by Smirnov et al. [11,116] from FeCl_3_ and 2-propyn-1-ol (propargyl alcohol, the simplest alcohol with an alkyne group). The deposition temperature was 200 °C, but no other experimental details were given. The focus was on the magnetic properties of the films: Uncompensated antiferromagnetism was observed.

Nilsen et al. [13] mention the use of nona-1,9-diol (1,9-nonanediol) together with TMA at 200 °C, but besides a QCM figure there is no further information regarding the system. In the same article TiCl_4_ and ED were used to fabricate hybrid thin films. Only a QCM graph acquired at deposition temperature of 75 °C and a cautioning to use long pulse lengths were given. They also combined 4-aminobenzoic acid with TiCl_4_ hoping that the carboxylic group would enhance the air stability of the hybrid film. No information regarding the depositions of these films was given but a QCM graph obtained at the deposition temperature of 200 °C.

The deposition strategy consisting of four stages by Chen et al. [49] has been also used to fabricate Al-containing hybrid films from TMA. The organic precursors were the same as with the Ti-containing counterparts, i.e., aminoethanol and propanedioyl dichloride.

Abdulagatov et al. [105] conducted pyrolysis studies on several hybrid films grown by using EG, GL, HQ and 2,3,5,6-tetrafluorobenzene-1,4-diol (tetrafluorohydroquinone) as the organic precursors: Al+GL, Al+EG, Al+HQ, Al+2,3,5,6-tetrafluorobenzene-1,4-diol, Zn+GL, Zn+HQ, Zr+EG, Hf+EG and Mn+EG were deposited at 150 °C [105]. Preliminary FTIR studies measuring 2,2-dimethoxy-1,6-diaza-2-silacyclooxetane+ethylene carbonate and TMA+3-buten-1-ol containing systems have been shortly mentioned [60].

In his review article, George [26] mentions polydimethylsiloxane films deposited by using H_2_O together with bis(dimethylamino)dimethylsilane, 1,3-dichlorotetramethyldisiloxane and dimethylmethoxychlorosilane. The GPC of the films was negligible after 15 deposition cycles, so depositions consisting of four pulsing steps, i.e., TMA+H_2_O+dimethylmethoxychlorosilane+H_2_O, at 200 °C were tried as well. Although this process yielded linear growth with GPC of 0.9 Å per cycle, the silicon content was too low. Hybrid films made by using TMA and 1,4-diazabicyclo[2.2.2]octane (triethylenediamine) are also shortly mentioned.

Tetrakis(dimethylamido)hafnium and EG have been combined to form hafnicones. Linear growth was observed at the deposition temperature of 145 °C. GPC decreased from 1.2 Å per cycle at 105 °C to 0.4 Å per cycle at 205 °C. The films were said to be very stable [28].

### Layer-engineered and nanostructured ALD/MLD films

Making various nanostructure and multilayer architectures by ALD and MLD is inherently easy due to the self-limiting cyclic growth process, which allows excellent control over the layer thickness and enables the conformal growth of the films. The ALD/MLD techniques have been successfully utilized to coat various nanostructures, such as nanoparticles, nanowires and carbon nanotubes. Both post-deposition annealing and wet-etching processes have been employed to remove the organic part of the inorganic–organic hybrid material, leaving porous oxide backbones. By adding a second material to the deposition process, it is also possible to make thin-film mixtures, superlattices and nanolaminates with tailored properties. In the following subchapters depositions of such nanostructured and layer-engineered ALD/MLD materials are discussed.

#### Fabrication of porous and nanostructured materials

**Figure 10:**
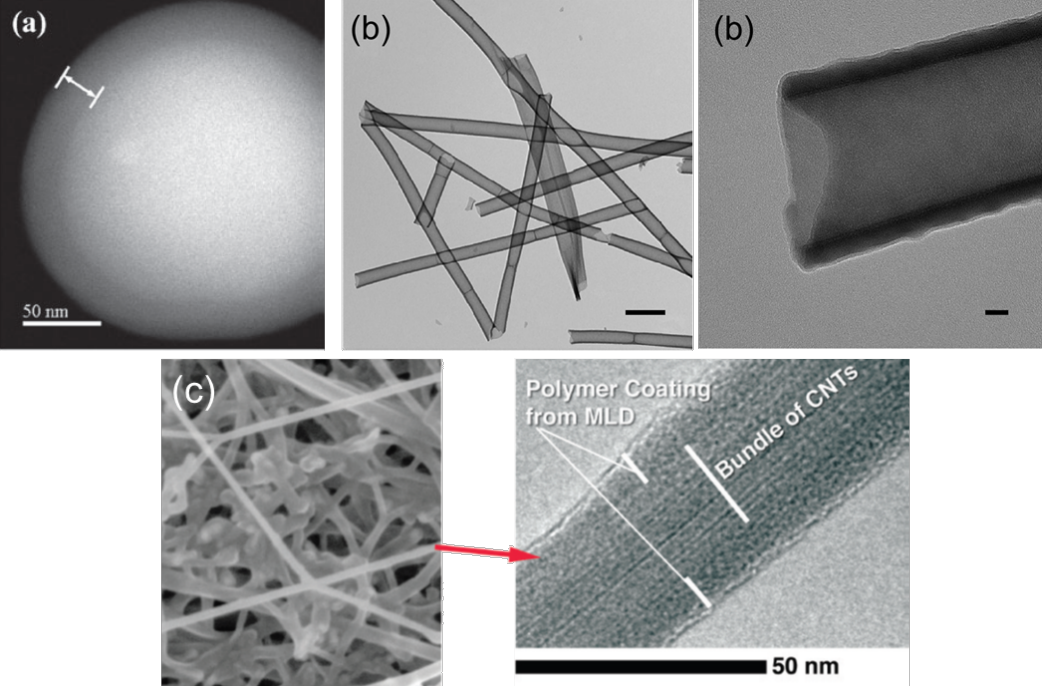
Electron microscope images of (a) 250 nm silica particles coated with a 25 nm thick layer of TMA+EG (reprinted with permission from [15], Copyright (2009) The Royal Society of Chemistry), (b) TMA+GLY film deposited on electro-spun polyvinyl alcohol fibers and annealed at 400 °C for 48 h (reprinted with permission from [38], Copyright (2011) American Chemical Society), and (c) metalcone coated carbon nanotubes (reprinted with permission from [95], Copyright (2013) American Chemical Society).

Hybrid ALD/MLD processes based on TMA and EG precursors have been used to coat nanoparticles and nanowires: TiO_2_ nanoparticles have been coated for photoactivity passivation [85], silica nanoparticles to fabricate Al_2_O_3_ films with controlled pore sizes (Figure 10a) [15,82], and CuO nanowires to obtain hollow Al_2_O_3_ nanostructures [83]. Hybrid TMA+EG coatings have also been utilized to fabricate Al_2_O_3_/zeolite composite membranes. In the latter example the organic constituent was removed by means of a post-deposition oxidation to form microporous Al_2_O_3_ on and between zeolite crystals. The membranes showed promise for H_2_ separation [89].

Liang et al. [98] deposited DEZ+EG coatings on titania nanoparticles. The DEZ+EG hybrid was observed to reduce the photoactivity of the nanoparticles. When the films were annealed in air, porous ZnO was formed. However, the number of pores was rather low. Moreover, the size of the pores fluctuated widely.

Porous TiO_2_ films have been obtained both by post-deposition annealing and by treating TiCl_4_+EG films with UV light [53,104]. After the post-deposition annealing the films were observed to have both amorphous and crystalline states and exhibit high photocatalytic activity. In a more recent paper by Abdulagatov et al. [105], GL was used together with TiCl_4_ to deposit thin films which then were annealed to form porous structures. For the annealed films a significantly reduced sheet resistance was achieved, being as low as 2.2 × 10^2^ Ω for films annealed at 800 °C; resistivity of the same films was 0.19 Ω·cm. These films are anticipated to have some potential in applications related to electrochemical reactions and electrochemical energy storage [105].

Gong et al. [38] deposited hybrid TMA+GLY coatings on electro-spun polyvinyl alcohol fiber mats, and subsequently annealed the samples at 400 °C for 48 h to remove the organic part. Conformality was verified by transmission electron microscopy, see Figure 10b. The pores formed were found to be mostly 5 Å micropores, with some mesopores [38]. Lee et al. [39] investigated the effect of the annealing temperature for similar types of TMA+GLY films and found that annealing at 350–500 °C removed the organic part of the films leaving Al_2_O_3_.

Hybrid TMA+GLY thin films have also been used together with ALD-grown ZnO and TiO_2_ to coat and protect electro-spun polyamide-6 (PA-6) nanofibers [90,93]. When TiO_2_ was used alone, the process caused fiber degradation. It was revealed that whereas a layer of ZnO did not offer sufficient protection from the following TMA+H_2_O treatment, when a layer TMA+GLY was deposited after ZnO, it prevented further degradation of the fibers when a TiO_2_ coating was applied on the top [90].

Recently, Brown et al. [95] coated carbon nanotubes with several different metalcone materials, including the TMA+EG, TMA+GL, TiCl_4_+GL, and DEZ+GL systems, see Figure 10c. The measurement of mechanical properties revealed that the MLD coatings caused a significant reduction in failure strain, a modest improvement in ultimate tensile strength, and a significant improvement in Young’s modulus. The greatest failure strain, about 3.9%, was achieved for TMA+GL coated nanotubes. The highest average Young’s modulus and the ultimate tensile strength were found for TMA+EG coated samples, with values of about 7 GPa and about 100 MPa, respectively. Young’s modulus for the uncoated carbon nanotube was 510 MPa, 2.2 GPa for 10 nm thick TMA+GL coated samples, and about 9 GPa for a composite coating consisting of 5 nm TMA+GL and 5 nm Al_2_O_3_.

Hybrid TMA+GLY films have also been grown on hydrophobic polydimethylsiloxane (PDMS) silicone exposed to sequential vapor infiltration of TMA+water. The aim of the study was to produce PDMS with a hydrophilic surface. When pure Al_2_O_3_ was used to coat the PDMS, a hydrophilic surface was obtained. However, after storing for 48 hours in ambient air the coating became more hydrophobic, losing the desired wetting characteristics. The TMA+GLY coating produced also a hydrophilic surface, which retained the hydrophilicity for more than two weeks in ambient air. However, the most hydrophilic and stable coatings were obtained by fabricating structures consisting of 100 cycles of Al_2_O_3_ and TMA+GLY [97].

Liang et al. [109] deposited TMA+tris(2-hydroxyethyl)amine+furan-2,5-dione thin films at 150 °C on 500 nm spherical silica particles. Carbon was removed from the films by soaking them in water for one day or by a 1 h oxidation in air at 400 °C. The resultant nanopores were 0.6–0.8 nm (and some 17 nm pores) and 0.8 nm in size, respectively.

Dry-etching with oxygen of TMA+EG and TMA+tris(2-hydroxyethyl)amine+furan-2,5-dione hybrids and wet-etching of TMA+tris(2-hydroxyethyl)amine+furan-2,5-dione with various solvents has also been studied. It was observed that HCl could be used to etch the thin film in a controlled manner, which makes of TMA+tris(2-hydroxyethyl)amine+furan-2,5-dione hybrid films promising materials for MEMS/NEMS applications [16].

Films from TiCl_4_, tris(2-hydroxyethyl)amine and propanedioyl dichloride have been fabricated on suspended CuO nanowires and carbon nanocoils by using a four-deposition-stage approach. The films were annealed at 600 °C in 5% H_2_/Ar for 2 h after the deposition. Excellent photocatalytic activity was observed for the nanoporous TiO_2_/carbon nanocoil structures. Nanoporous Al_2_O_3_ structures were also obtained using TMA as the inorganic precursor [49].

#### Fabrication of thin-film mixtures, superlattices and nanolaminates

The ALD/MLD thin-film mixtures, superlattices and nanolaminates are all made by using at least two different materials, for example a purely organic material and an oxide, by varying the number of deposition cycles of each material. The distinction between the various types of such layer-engineered materials is not completely unambiguous. In principle, a mixture is formed when the number of deposition cycles of each material is kept relatively low; then, if no full monolayer coverage is achieved with each material, the materials do not form separate layers but instead a homogenous mixture. On the other hand, if the growth happens in a well-controlled manner, repeated cycles may lead to a superlattice structure. The nanolaminates are formed by using larger number of deposition cycles, so that at least one of the materials achieves nanometer scale thickness and the materials form individual layers in the structure.

**Figure 11:**
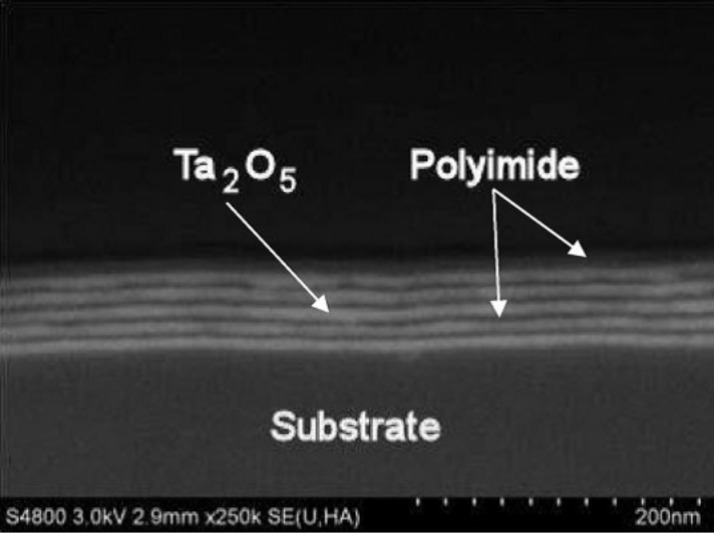
Field emission scanning electron microscopy image of a nanolaminate fabricated using five bilayers of 10 nm Ta_2_O_5_ and 10 nm polyimide (reprinted with permission from [62], Copyright (2009) WILEY-VCH Verlag GmbH & Co. KGaA, Weinheim).

Only three articles published so far feature multilayer structures that contain purely organic nanoscale layers [62,70,72]. Salmi et al. [62] fabricated Ta_2_O_5_/polyimide nanolaminate structures consisting of nanoscale inorganic Ta_2_O_5_ and organic polyimide layers. Tantalum ethoxide and water were used to deposit Ta_2_O_5_, and PMDA and hexane-1,6-diamine for the polyimide deposition. All nanolaminates were constructed from five bilayers, with three different constructions: 10 nm Ta_2_O_5_ + 10 nm polyimide (shown in Figure 11), 15 nm Ta_2_O_5_ + 5 nm polyimide, and 5 nm Ta_2_O_5_ + 15 nm polyimide. The study focused on dielectric and mechanical properties of these nanolaminates. It was observed that the permittivity of the nanolaminates increased with the Ta_2_O_5_ content, but not in a linear manner. The trend was similar for the refractive index, but in a more linear way. It was also seen that the insulating properties of the parent materials could be improved through the fabrication of nanolaminate structures. Nanointendation measurements revealed that the softness, elasticity and plasticity of the films increased with increasing polyimide content.

Loscutoff et al. [70] deposited purely organic nanolaminates at room temperature consisting of 30–50 nm thick PDIC+ED and PDIC+2-[(2-aminoethyl)sulfanyl]ethanamine layers. The growth of the latter material was not discussed in detail in the article, though. Depth profiles by a sputter technique were investigated on two different nanolaminates: The first consisted of two layers of 40 nm PDIC+ED, with a 50 nm thick PDIC+2-[(2-aminoethyl)sulfanyl]ethanamine layer in between, the second of two 30 nm thick PDIC+2-[(2-aminoethyl)sulfanyl]ethanamine layers with a 40 nm thick PDIC+ED layer between them. Although a small unexpected sulfur signal indicated the presence of some unreacted sites, it could be concluded that the composition of the nanolaminates agreed well with the expected values.

As part of their study on potential photoresist materials, Zhou et al. [72] also investigated nanolaminates of purely organic constituents, i.e., PDIC+ED and PDIC+2,2-(propane-2,2-diylbis(oxy))diethanamine. Organic films of the former type are stable in HCl, whereas of the latter type are not. Three-layer structures were deposited to investigate the stability of the nanolaminates in HCl: First 3, 6 or 9 cycles of PDIC+ED were deposited, followed by 3 or 6 cycles of PDIC+2,2-(propane-2,2-diylbis(oxy))diethanamine, finished with 3 cycles of PDIC+ED on the top. According to the thickness measurements, only the bottom layer remained after the acid treatment, indicating that the cleaving occurred at the positions of acid-labile groups. Also nanolaminates consisting of 3 cycles of PDIC+2,2-(propane-2,2-diylbis(oxy))diethanamine and 8 or 15 cycles of PDIC+ED on top were deposited. These films were soaked with triphenylsulfonium triflate, UV radiated, baked and developed. No significant changes in thickness were observed and it was concluded that the top layer behaves as a photoacid generator barrier.

Quite many inorganic–organic hybrid compositions have been utilized to fabricate homogeneous thin-film mixtures, superlattice structures and nanolaminates. Lee et al. [86–87] fabricated mixtures consisting of TMA+EG alucone and Al_2_O_3_ at 135 °C, and varied the oxide:hybrid cycle ratio from 1:3 to 6:1 to accurately control the density, refractive index, elastic modulus and hardness of the films. The values obtained varied between those for pure alucone and pure Al_2_O_3_, for density from 1.6 to 3.0 g/cm^3^, for refractive index from 1.45 to 1.64, for elastic modulus from 20 to 200 GPa, and for hardness from 1 to 13 GPa.

The stability and optical properties of mixtures of alucone (from TMA+EG) and Al_2_O_3_ have been also studied and compared to those of pure alucone films. The depositions were carried out on Si(100) substrates at 150 °C. It was observed that when the sample was kept in air the thickness and refractive index of pure alucone decreased by about 20% and about 1.4%, respectively, over the first three days, after which there were no further changes. When the alucone films were kept in a desiccator, there were no significant changes in thickness or refractive index. It was also revealed that capping a 100 nm thick alucone layer with a 20 nm thick Al_2_O_3_ layer improved the stability, as did the fabrication of a nanolaminate structure consisting of five bilayers of 20 nm of alucone and 10 nm of Al_2_O_3_. When homogeneous alucone:Al_2_O_3_ mixtures were investigated, the mixture with a ratio of 5:1 lost 12–17% of its initial thickness in open air, whereas the 5:5 mixture was stable, and the thickness of the 1:1 mixture was reduced by 4%. The change in refractive index for the 5:1 mixture was larger than for the pure alucone film. Measurements performed on annealed films showed that the refractive index could be tuned by the speed of the annealing: Slow annealing resulted in a film with more pores and less collapse in the film. When heated, the 1:1 mixture showed numerous cracks while the 5:1 mixture showed better resistance to the heat treatment. When the 5:1 mixture was heated with 10 °C/h, the refractive index dropped from the initial 1.52 to 1.34 while the thickness decrease was about 28%. In case of the 5:5 mixture heating did not destroy the film, but the refractive index decreased to 1.44 [92].

Zhou et al. [34] deposited mixtures consisting of TMA+1,4-diaminobenzene hybrid and Al_2_O_3_ with the hybrid:oxide ratios of 1:1, 1:2, 1:3 and 1:4. The 1:4 mixture showed improved stability in ambient air: The film thickness decreased less than 5% when kept at ambient air for several tens of weeks. The mixtures showed excellent electrical insulating properties. A current density of 2.3 × 10^−8^ A·cm^−2^ at an electric field of 1 MV·cm^−1^, and a dielectric constant of about 6.2 were measured for the 1:4 mixture. The 1:4 mixture showed clear charge trapping behavior, but not good enough to be used as a charge trap layer for a charge trap flash memory.

**Figure 12:**
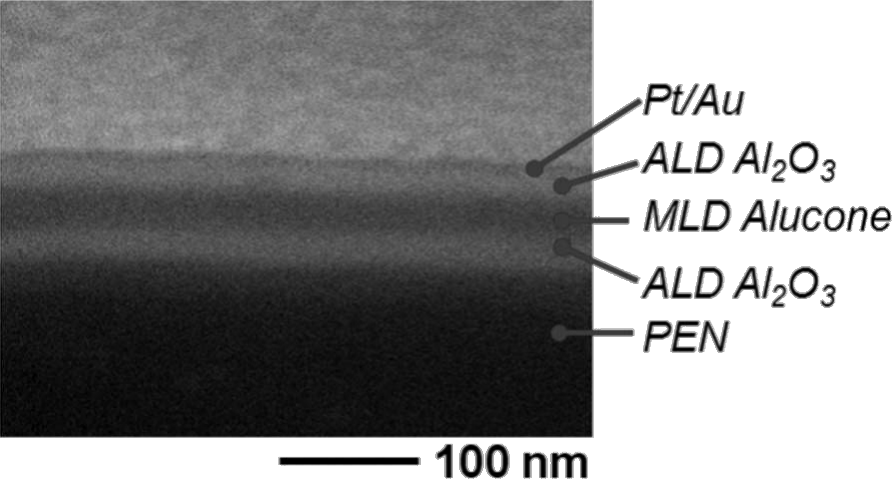
A nanolaminate coating consisting of Al_2_O_3_ and TMA+EG alucone layers with targeted thicknesses of 25 and 15 nm, respectively, on a polyethylene naphtalate (PEN) substrate (reprinted with permission from [81], Copyright (2009) American Institute of Physics).

Miller et al. [81] investigated the mechanical properties of Al_2_O_3_/TMA+EG/Al_2_O_3_ nanolaminates grown at 155 °C on polyethylene naphtalate substrates. The layer thicknesses were 10/3/10 nm, 25/15/25 nm (shown in Figure 12), 25/3/25 nm, and 25/192/25 nm. The nanolaminates exhibited reduced critical strains at fracture when compared to pure components. This was attributed to the low toughness of the TMA+EG alucone [81]. According to the microcantilever-facilitated curvature studies done later the curvature for the pure TMA+EG hybrid evolved when the films were stored in ambient air for two weeks. Investigation on 25/192/25 nm thick Al_2_O_3_/TMA+EG/Al_2_O_3_ nanolaminates showed that the topmost Al_2_O_3_ layer stabilized the structure, possibly by shielding the underlying TMA+EG layer from moisture [84].

Nanolaminates fabricated using TMA+EG and Al_2_O_3_ were also investigated by Vähä-Nissi et al. [88]. The depositions were carried out at 100 °C on biopolymer (biaxially oriented polylactic acid) substrates, and the samples were characterized for their oxygen transmission rate (OTR) and water vapor transmission rate (WVTR). It was shown that the five-layer nanolaminates investigated, i.e., Al_2_O_3_/TMA+EG/Al_2_O_3_/TMA+EG/Al_2_O_3_, worked essentially better than pure Al_2_O_3_ films as gas-barrier coatings on biopolymer materials. Purely inorganic coatings are somewhat brittle (as clearly evidenced from SEM images revealing large cracks for pure Al_2_O_3_ films on top of flexible biopolymer substrates), and straining them leads to defects and deteriorated gas-barrier properties. Apparently the intervening hybrid layers improve the flexibility of the coating. For the laminates the defect concentration was found to be considerably smaller compared to Al_2_O_3_ films after mechanical straining. The best barrier properties were achieved for the five-layer laminate deposited with 50 cycles of both components. The laminates were also found to be stable in air which was not the case for a thin Al_2_O_3_ layer alone on the biopolymer substrate.

The WVTR of TMA+EG and Al_2_O_3_ coatings was investigated also by Park et al. [91]. The films were deposited at 85 °C on polyethylene naphthlate substrates. The WVTR values measured at 85 °C and 85% relative humidity for alucone, Al_2_O_3_ and Al_2_O_3_/alucone nanolaminate coatings were ca. 1.1, 0.037 and 0.021 g/(m^2^·day), respectively. The improved WVTR value of the nanolaminate coating when compared to the pure alucone and oxide layers alone was attributed to a synergy effect: As the alucone layer reacts readily with water, it reduces the diffusion speed as well as increases the diffusion paths of the water vapor.

The Zn-based hybrid DEZ+HQ has been combined with ZnO to form mixtures which were anticipated to show enhanced electrical conductivity due to electron band overlap between the π-electrons of the HQ ring and ZnO [98–99]. The pure ZnO film had a conductivity of 14 S/cm in ambient light, whereas the pure hybrid film was nonconductive. The 1:1, 1:3 and 2:2 mixtures of ZnO:DEZ+HQ had higher electrical conductance than ZnO, i.e., 116, 40 and 170 S/cm, respectively, whereas the 1:5 and 5:5 mixtures exhibited lower conductance than ZnO, i.e., 6 and 13 S/cm. respectively. The elastic modulus varied depending on the precise composition of the mixtures, being about 30 GPa for a pure DEZ+HQ hybrid film, about 50 GPa for a 1:5 mixture, about 120 GPa for a 1:1 mixture, and about 150 GPa for a pure ZnO film. Also the hardness of the films was affected by the moiety concentration, being about 1, 4, 5 and 13 GPa for the same films, respectively. Pure DEZ+HQ, the 1:1 mixture and the pure ZnO were all highly transparent in visible spectrum [99].

Superlattice structures have been successfully fabricated where nondoped or Al-doped ZnO layers of nanometer-scale alternate with extremely thin organic (hydroquinone) layers. The films were deposited with the DEZ+H_2_O, TMA+H_2_O and DEZ+HQ processes at 220 °C, with the oxide:hybrid deposition cycle ratio varying from 199:1 to 1:1 [94,102–103]. FTIR and XRR studies confirmed the incorporation of organic layers and the formation of superlattice structures, respectively. When heated for one hour up to 700 °C, no changes in XRR patterns of the (Zn,Al)O:HQ films were observed until 450 °C, after which coarsening of the film started to inflict noise. The superlattice-originated XRR peaks were still present when annealed up to 650 °C. No visual change in the films was observed until heat-treated at 700 °C. Apparently the ZnO layers protect the underlying organic layers from decomposition at elevated temperatures. The eventual goal of the study was to suppress lattice thermal conductivity and/or enhance the Seebeck coefficient of ZnO films through the introduction of intervening organic layers such that the superlattice films could show overall enhanced thermoelectric characteristics, i.e., concomitant large Seebeck coefficient, high electrical conductivity and low thermal conductivity. Preliminary characterization of the (Zn,Al)O:HQ superlattice films confirmed that the films indeed showed promise as thermoelectric materials [94]. More recently it was demonstrated that similar superlattice structures consisting of thicker layers of ZnO combined with individual organic layers could be made not only with HQ but also with an AP or ODA layer [103]. The ZnO:hybrid ratio varied between 199:1 and 39:1, and the superlattice structure was confirmed by XRR. Resistivity and Seebeck coefficient measurements showed an increase in carrier concentration for small concentrations of organic layer, whereas at higher concentrations a large reduction in carrier concentration was observed.

Liu et al. [101] fabricated oxide-hybrid mixtures by using the same DEZ+H_2_O and DEZ+HQ processes as described above for the ZnO:HQ superlattices, and determined the thermal conductivity for the 1:1 mixture at about 0.13–0.15 W/(m·K). This value is much lower than what was measured by the same group for pure DEZ+HQ hybrid and discussed earlier in this review. It was suggested, that when employing structurally vastly different oxide and hybrid constituents in the material fabrication, the ZnO flakes and hybrid chains scatter efficiently phonons, resulting in a reduced thermal conductivity. The volumetric heat capacity for the 1:1 mixture was about 2.9 J/(cm^3^·K), being only slightly less than what was reported for the DEZ+HQ system.

The DEZ+AP and DEZ+H_2_O processes have been combined to form mixtures and nanolaminates. The crystallinity and density of the mixtures were varied by the number of hybrid and oxide cycles during the depositions. Although the hybrid containing films were unstable in air, which made the AFM measurements somewhat inaccurate, adding amorphous hybrid to crystalline ZnO had a smoothing effect on the samples. The nanolaminates which had a minimum of 3 nm thick ZnO layer on top were stable in ambient air and had a rather constant RMS roughness of 1–1.4 nm. According to the nanointendation measurements the DEZ+AP hybrid was soft, with a contact modulus of a typical polymer. Although ZnO is also soft, it is still harder and stiffer than the pure hybrid. Adding ZnO to the hybrid was observed to have little effect up to 1:1 hybrid:oxide ratio. From the nanointendation measurements performed on nanolaminates it was concluded that the thicker the ZnO layer is, the more it enhances the hardness of the film and a thin ZnO layer can actually reduce film strength for some unknown reason. Wet-etching tests showed that adding ZnO does now improve the chemical stability of the mixtures. However, acetone treatment was observed to remove only the organic part of the film, leaving a porous oxide backbone [106].

Mixtures of TiO_2_ and TiCl_4_+ODA have been deposited with oxide:hybrid ratios from 1:1 to 10:1. A good control over the RMS roughness value and refractive index was achieved by varying the mixture composition. Also the density, reduced modulus, hardness and crystallinity of the material systematically depended on the oxide:hybrid ratio. Wet-etching tests carried out with several solvents indicated that the mixtures were chemically extremely stable [113].

For ZrO_2_:ZTB+EG mixtures in which the oxide:hybrid ratio was varied from 1:3 to 3:1 [54,87], it was found that the refractive index and elastic modulus changed continuously from values for pure ZTB+EG (*n* ≈ 1.63 and *E* ≈ 27 GPa) to those of pure ZrO_2_ (*n* ≈ 1.86 and *E* ≈ 97 GPa). Also density and hardness varied according to the oxide:hybrid ratio.

Superlattice structures consisting of TiO_2_ and TiCl_4_+HDD were successfully deposited at 100 °C by repetition of 50 cycles of TiO_2_ and one cycle of TiCl_4_+HDD. TEM images showed individual oxide and hybrid nanolayers. The films remained stable when annealed up to 400 °C [55].

As 7-OTS is a heterobifunctional material, for which the terminal C=C group is required to be converted to a carboxylic group by ozone treatment during the growth process, the 7-OTS hybrid materials grow in extremely well controlled layer-by-layer manner. Hybrid films based on 7-OTS have been used in several studies to fabricate various superlattice and nanolaminate structures. Four bilayers consisting of 1.1 nm thick 7-OTS+Ti(OCH(CH_3_)_2_)_4_ hybrid and 2 nm thick TiO_2_ were used to fabricate nanolaminates. The nanolaminate was then sandwiched between Al metal wires to study the electrical properties of the hybrid-oxide material. The constructed device had a large endurance and a long retention time, which demonstrated the potential application of the nanolaminates as nonvolatile memory materials [10].

Investigations on multilayered structures of 7-OTS+Ti(OCH(CH_3_)_2_)_4_ and TiO_2_ deposited on PEN indicated that the TiO_2_ blocked water permeation. The WVTR experiments suggested that the lag time for the structures was extended due to the tortuous path effect and water accumulation in the organic layers. For a structure consisting of five bilayers a WVTR value of 7.0 × 10^−4^ g/(m^2^·day) during a lag time of 155 h at 60 °C and a relative humidity 85% was obtained [40].

Superlattices with hybrid-Al_2_O_3_-hybrid-TiO_2_ structures with various mixing ratios have been fabricated using 7-OTS+Ti(OCH(CH_3_)_2_)_4_ and 7-OTS+TMA. The formed structures when annealed were stable up to about 500 °C. The coatings showed good flexibility, were mechanically stable, and had various unique electrical properties. Organic pentacene thin-film transistors fabricated by using the superlattices on flexible plastic substrate had a drain current of 1.5 μA, a field effect mobility of 0.54 cm^2^/(V·s), and an inverter voltage gain −d*V*_out_/d*V*_in_ ≈ 4.5 when operated at a voltage of −2 V [41].

A non-volatile flash memory thin-film transistor was made using 7-OTS+Ti(OCH(CH_3_)_2_)_4_ and 7-OTS+TMA layers between ZnO and pentacene. The device showed promising non-volatile memory effects when operated at low voltages [42]. Organic pentacene thin-film transistors were also fabricated by using 7-OTS+ZTB and ZrO_2_. A maximum field effect mobility of 0.63 cm^2^/(V·s) was measured, when operating at −1 V with an on/off current ratio of about 10^3^ [43].

Nanolaminates consisting of 7-OTS+DEZ and ZnO layers have also been deposited. The thin-film transistors made by using the nanolaminate had a high field effect mobility of 7 cm^2^/(V·s), when operating at 3 V with an on/off current ratio of 10^6^ and with a threshold voltage of 0.6 V. It was also concluded that the 7-OTS+DEZ provides structural flexibility in the superlattice [44].

**Figure 13:**
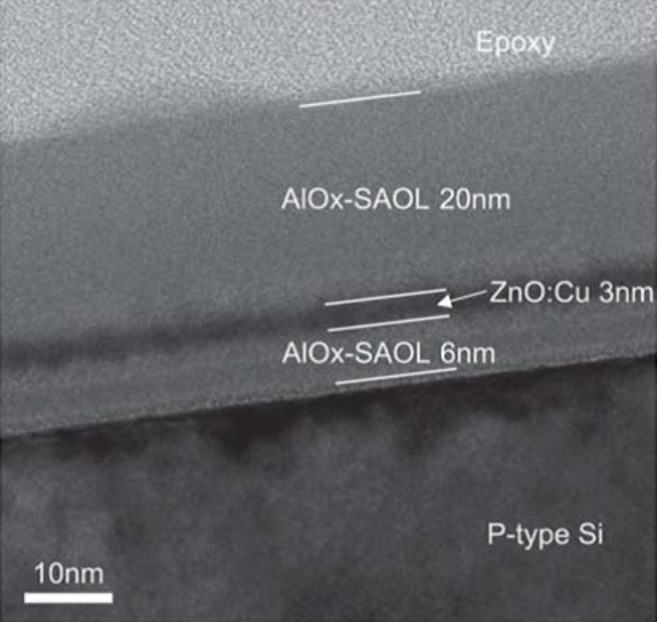
An HRTEM image of a capacitor memory device fabricated by using Al-containing hybrid (marked as AlOx-SAOL) as blocking and tunneling layers with a ZnO:Cu charge trap layer in between (reprinted with permission from [46], Copyright (2012) The Royal Society of Chemistry).

Han et al. [46] fabricated floating-gate nonvolatile memory transistors from two types of hybrid layers: Al-containing hybrid layers were deposited by using 7-OTS, water, O_3_ and TMA as precursors, whereas DEZ and HDD were used for the Zn-containing layers. Capacitor memory devices constructed by using Al-containing hybrid as blocking and tunneling layers with ZnO:Cu charge trap layer sandwiched between them (Figure 13), had a large memory window of 14.1 V operated at ±15 V. The same structure was then used together with a Zn-containing hybrid as a semiconducting layer to form nonvolatile memory transistors which operated in voltage range of −1 to 3 V. The high writing/erasing (+8 V/−12 V) current ratio of 10^3^ obtained with the device indicated that the tested construction showed promise for memory electronics applications.

## Conclusion

The application perspectives of the original ALD thin-film technology have become considerably wider through the introduction of purely organic moieties as building units in the chemical atomic-scale controlled deposition process. Now by taking advantage of the MLD technique we are not only able to materialize in a molecular layer-by-layer manner high-quality thin films of various commercially attractive organic polymers but also of inorganic–organic hybrid materials, potentially combining the best attributes of the two entirely different chemistries.

The organic polymers made up till now by means of the MLD technique include various amides, imides, imide–amides, ureas, urethanes, esters and imines, while in the case of the ALD/MLD-grown inorganic–organic hybrid thin films the metal species variety covers the elements Al, Zn, Ti, Zr, Hf, V, Si, Fe and Mn. Although a rapidly increasing number of different precursors have already been exploited, the field is nothing but just approaching its emergent stage. Nevertheless essentially ideal MLD and ALD/MLD processes have already been developed for several precursor combinations such that the film growth rates achieved well correspond to the values calculated on the bases of the expected lengths for straight polymer chains. Examples of such ideally behaving processes are the purely organic hexanedioyl dichloride+hexane-1,6-diamine and heptane-1,7-diamine+nonanedioyl dichloride systems, and the hybrid diethylzinc+hexa-2,4-diyne-1,6-diol, TiCl_4_+hexa-2,4-diyne-1,6-diol and TiCl_4_+4-aminophenol systems.

The layer-by-layer manner in which the films are grown in both ALD and MLD, provides us yet another powerful means of fine-tuning the film properties by depositing on-demand designed thin-film mixtures, superstructures and nanolaminates. Optical and mechanical properties, surface roughness and degree of crystallinity have been successfully tuned by mixing different deposition cycles, whereas control over the chemical stability, electrical and gas-barrier properties and electrical and thermal conductivities has been achieved by constructing well-defined superlattice and nanolaminate structures. Post-deposition treatments of films containing organic moieties have also proven to further expand the application range of the ALD/MLD fabricated hybrid thin films as such treatments enable, e.g., to produce porous coatings.

The work on the ALD/MLD grown organic and inorganic–organic thin films is still in its beginning phase. Deeper studies are definitely required to shed light even on the precise growth mechanisms of these fundamentally new types of thin films, hopefully giving us better insight to select new well-behaving precursor pairs. As the huge potential of the hybrid films has been recognized, the number of articles featuring properties related to specific applications keeps rising. Recently, it was for example demonstrated that periodically repeating organic layers embedded in thicker inorganic layers can efficiently block heat conduction. This result is highly promising in the field of thermoelectrics. Tunable refractive index should on the other hand be extremely important for optical applications. As another example, nanolaminate structures from oxides and hybrids improve the gas barrier properties of the protective coatings. Especially noteworthy are also the porous structures, which could be used in optics, electronics and catalysis, to name just a few examples. In short, the prospects of the ALD/MLD fabricated films are excellent.
